# Single-atom Sn-Zn pairs in CuO catalyst promote dimethyldichlorosilane synthesis

**DOI:** 10.1093/nsr/nwz196

**Published:** 2019-11-28

**Authors:** Qi Shi, Yongjun Ji, Wenxin Chen, Yongxia Zhu, Jing Li, Hezhi Liu, Zhi Li, Shubo Tian, Ligen Wang, Ziyi Zhong, Limin Wang, Jianmin Ma, Yadong Li, Fabing Su

**Affiliations:** 1 Gripm Advanced Materials Co., Ltd, Beijing 101407, China; 2 State Key Laboratory of Multiphase Complex Systems, Institute of Process Engineering, Chinese Academy of Sciences, Beijing 100190, China; 3 Zhongke Langfang Institute of Process Engineering, Langfang 065001, China; 4 Beijing Key Laboratory of Construction Tailorable Advanced Functional Materials and Green Applications, School of Materials Science and Engineering, Beijing Institute of Technology, Beijing 100081, China; 5 Department of Chemistry, Tsinghua University, Beijing 100084, China; 6 College of Engineering, Guangdong Technion–Israel Institute of Technology (GTIIT), Shantou 515063, China; 7 Technion–Israel Institute of Technology (IIT), Haifa 32000, Israel; 8 School of Physics and Electronics, Hunan University, Changsha 410082, China; 9 Institute of Industrial Chemistry and Energy Technology, Shenyang University of Chemical Technology, Shenyang 110142, China

**Keywords:** dual single-atom promoters, CuO catalyst, dimethyldichlorosilane synthesis, Rochow reaction, catalytic performance

## Abstract

Single-atom catalysts are of great interest because they can maximize the atom-utilization efficiency and generate unique catalytic properties; however, much attention has been paid to single-site active components, rarely to catalyst promoters. Promoters can significantly affect the activity and selectivity of a catalyst, even at their low concentrations in catalysts. In this work, we designed and synthesized CuO catalysts with atomically dispersed co-promoters of Sn and Zn. When used as the catalyst in the Rochow reaction for the synthesis of dimethyldichlorosilane, this catalyst exhibited much-enhanced activity, selectivity and stability compared with the conventional CuO catalysts with promoters in the form of nanoparticles. Density functional theory calculations demonstrate that single-atomic Sn substitution in the CuO surface can enrich surface Cu vacancies and promote dispersion of Zn to its atomic levels. Sn and Zn single sites as the co-promoters cooperatively generate electronic interaction with the CuO support, which further facilitates the adsorption of the reactant molecules on the surface, thereby leading to the superior catalytic performance.

## INTRODUCTION

As a result of their maximum atom utilization and unique electronic properties, single-atom catalysts have shown superior catalytic properties in a wide variety of reactions compared to conventional nanoparticle catalysts [1–9]. Thus they have received increasing research interest in recent years. At present, the stabilization of these single atoms under harsh reaction conditions, such as elevated temperatures and pressures [10–12], is still the main concern but can potentially be well-addressed by making use of uniform defects of underlying supports as anchoring sites [13]. The defects, like dopants and atom vacancies, also have the potential to alter the coordination environment and charge distribution on the surface [14], thus further improving the catalytic activity. To our knowledge, in recent years, the primary attention has been paid to the surface oxygen vacancies on supports [13,15,16], while the research activities on how to make use of cation vacancies to ensure stable anchoring are still not enough.

On the other hand, promoters, which can further enhance the catalytic performances of many catalysts and are therefore of great importance in catalysis [17–19], are rarely studied in their single-atom forms for catalytic reactions. Therefore, preparing catalysts with single-sited promoters that possess similar advantages to single-atom catalysts, such as the structural simplicity and homogeneity [20], should be of great interest. These single-sited promoters may not only help to elucidate their real promotion mechanism in catalytic reactions, but also open up a new path to optimize catalyst performance. For instance, Wang *et al.* [21] reported that incorporating single-site Sn on TiO_2_ as the promoter could create more oxygen vacancies on its surface, leading to the improved catalytic activity and selectivity in nitroarene hydrogenation. Very recently, it has been demonstrated that the doping of CeO_2_ with single-atom Ni as the promoter is an effective means to generate oxygen vacancies, which promote the selective hydrogenation of acetylene to ethylene [22]. Because two or more kinds of promoters are often used in one industrial catalyst [17,23], the exploration of the preparation of two single-site promoters and the synergistic effect between them on catalytic reaction is of great interest in catalysis. However, due to the difficulty in the synthesis, there has been no such report so far.

Here we report the synthesis of a new catalyst consisting of atomically dispersed Sn and Zn co-promoters on the CuO surface (denoted as Zn_1_-Sn_1_/CuO, where ‘1’ represents the single atom, and the same is applied hereafter). Direct experimental evidence shows that single-site Sn is incorporated into the lattice of CuO catalysts to generate Cu^2+^ vacancy sites, which further serve as anchoring sites to stabilize single-site Zn. Density functional theory (DFT) calculations also show that on the Sn-doped CuO(110) surface, the formation energy of Cu vacancy is 0.78 eV lower than that on the clean CuO(110), which indicates it is easier to form Cu vacancies in the Sn-doped surface. This novel Zn_1_-Sn_1_/CuO catalyst displays excellent activity, selectivity and stability, much higher than that of the traditionally prepared catalysts where the promoters exist in the form of nanoparticles in the synthesis of dimethyldichlorosilane via the Rochow reaction. The DFT calculations further reveal that the atomically dispersed Sn and Zn on the CuO surfaces can enhance the adsorption of reactant molecules on the surface compared to clean CuO, which contributes to the excellent catalytic properties of the former.

## RESULTS AND DISCUSSION

### Synthesis and characterization of Zn_1_-Sn_1_/CuO catalyst

**Figure 1.**
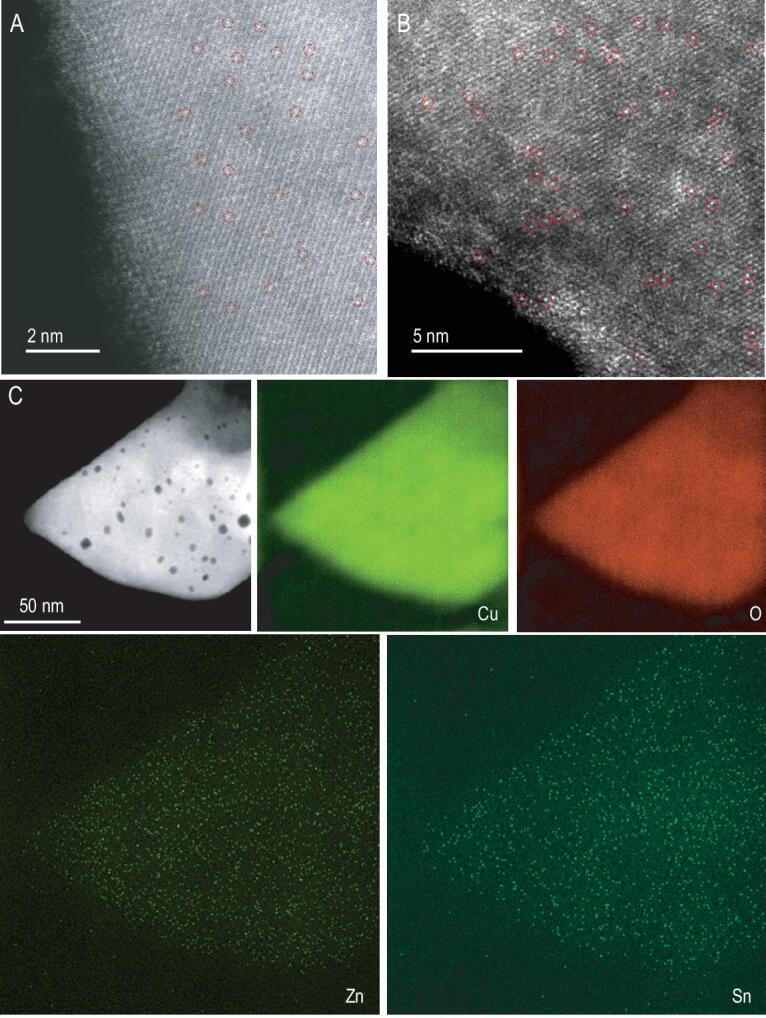
(A) AC HAADF-STEM image of Sn_1_/CuO. (B) AC HAADF-STEM and (C) HAADF-STEM images as well as the corresponding EDS mappings of 0.1Zn_1_-Sn_1_/CuO. The bright dots marked with the red circles in (A) and (B) indicate the single atom.

CuO and Sn_1_/CuO were synthesized via a facile hydrothermal treatment according to the previously reported method with some modifications [24]. Both the samples exhibit morphologies of nanosheets with an average thickness of about 600 nm (Fig. S1 in the online supplementary material). X-ray diffraction (XRD) patterns (Fig. S2 in the online supplementary material) show that, compared with those of pure CuO, the diffraction peaks of Sn_1_/CuO slightly shift to higher angles. Furthermore, no peaks of Sn species are detected in Sn_1_/CuO, indicating that Sn has been doped into the lattice of CuO and is highly dispersed [25]. This phenomenon is because the isomorphous substitution of Cu ions with Sn atoms having a smaller ionic radius would lead to a lattice contraction. It is further verified by a high-resolution transmission electron microscopy (HRTEM) image (Figs S3 and S4 in the online supplementary material), which does not show any lattice corresponding to Sn species [26], but does show the formation of numerous holes on the surface of Sn_1_/CuO (marked by the red circles). Furthermore, it is found that the brighter spots in the image (Fig. 1A) represent Sn atoms, which are identified by using *Z* contrast in aberration-corrected high-angle annular dark-field scanning transmission electron microscopy (AC HAADF-STEM) as the atomic numbers of Sn and Cu are *Z* = 50 and 29, respectively, which are noticeably different [27]. Subsequently, a simple wet impregnation method [28] was employed to anchor the single-site Zn on defective-rich Sn_1_/CuO to form *x*Zn_1_-Sn_1_/CuO (*x* refers to weight ratios of Zn to CuO; detailed information of the synthesis is shown in the online supplementary material). As shown in Fig. S5 in the online supplementary material, the synthesized *x*Zn_1_-Sn_1_/CuO maintains the morphology of Sn_1_/CuO, and there is no obvious change in size. Transmission electron microscope (TEM) and HRTEM images (Figs S6–S8 in the online supplementary material) reveal no formation of any visible clusters or nanoparticles in *x*Zn_1_-Sn_1_/CuO, and show crystalline lattice spacings of 0.27 nm corresponding to the (110) plane of CuO, which supports the high dispersion of Zn atoms. Also, we can see that there are no additional diffraction peaks of Zn species in the corresponding XRD patterns (Fig. S9 in the online supplementary material), suggesting that Zn is in the form of a highly dispersed state as well [29]. The AC HAADF-STEM image, shown in Fig. 1B, clearly displays abundant small bright dots, which should be assigned to Sn atoms. It is because the Zn atom cannot be identified owing to the very close atomic numbers of Zn (*Z* = 30) and Cu (*Z* = 29). HAADF-STEM energy-dispersive X-ray (EDX) elemental mapping (Fig. 1C) further confirms the presence of Zn, Sn, O and Cu elements in 0.1Zn_1_-Sn_1_/CuO. The EDX spectra of this sample also show the peaks of Sn and Zn (Fig. S10 in the online supplementary material). Therefore, it can be concluded that both Sn and Zn present as single atoms distributed homogeneously throughout the CuO catalyst. The contents of Zn and Sn measured by EDX (the inset of supplementary Fig. S10) and inductively coupled plasma optical emission spectrometry (ICP-OES) analysis are basically consistent with the feeding ratios of the metal precursors in the synthesis (Table S1 in the online supplementary material) [30].

**Figure 2.**
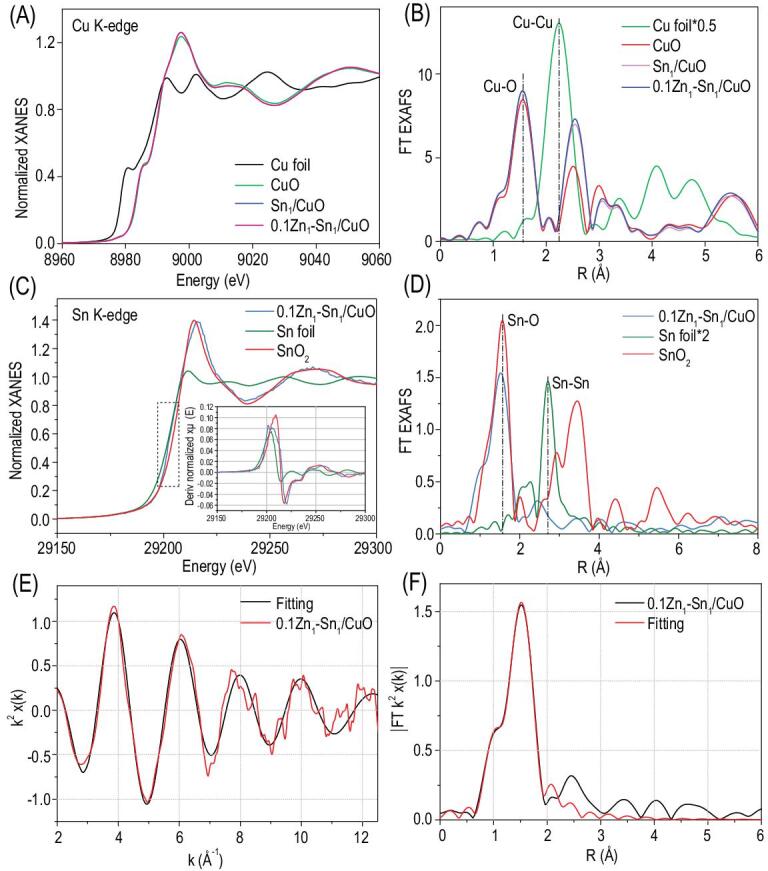
(A) The normalized Cu K-edge XANES spectra; (B) FT k^2^-weighted EXAFS spectra of Cu; (C) the normalized Sn K-edge XANES (inset is the deriv image) spectra; (D) FT k^2^-weighted EXAFS spectra of Sn; (E) k space EXAFS spectrum of the 0.1Zn_1_-Sn_1_/CuO at the Sn K-edge; and (F) corresponding FT-EXAFS fitting curves of 0.1Zn_1_-Sn_1_/CuO.

The coordination states and local structure of Sn and Cu were investigated by X-ray absorption spectroscopy (XAS) analysis. It should be noted that owing to the extremely low amount of Zn relative to Cu, the signal of Zn is shielded by that of Cu, and thus it is hard to obtain the structure information of Zn. As shown in Fig. 2A, the Cu K-edge X-ray absorption near edge structure (XANES) spectra suggest that there is no noticeable change in the valence state of Cu in Sn_1_/CuO and 0.1Zn_1_-Sn_1_/CuO as compared with that of CuO. The Fourier transform extended X-ray absorption fine structure (FT-EXAFS) curves (Fig. 2B) show that all the samples of CuO, Sn_1_/CuO and 0.1Zn_1_-Sn_1_/CuO exhibit one main peak at 1.56 Å, which is ascribed to the contribution of the first shell of Cu-O. However, compared with that in CuO, the peak at about 2.50 Å attributed to the second shell of Cu-O contribution is obviously enhanced in both Sn_1_/CuO and 0.1Zn_1_-Sn_1_/CuO, suggesting the change of the local environment of Cu in these two samples. As shown in Fig. S11 in the online supplementary material, and Fig. 2C, the Sn K-edge XANES spectra of Sn_1_/CuO and 0.1Zn_1_-Sn_1_/CuO are located between those of Sn foil and SnO_2_ references, revealing that the isolated Sn atoms are partially positively charged. The FT curves of EXAFS of Sn_1_/CuO (Fig. S12A in the online supplementary material) and 0.1Zn_1_-Sn_1_/CuO (Fig. 2D) present only an Sn-O peak at about 1.52 Å, and no Sn-Sn peak at 2.71 Å is detected, clearly suggesting that Sn atoms are atomically dispersed and coordinated by oxygen atoms in both Sn_1_/CuO and 0.1Zn_1_-Sn_1_/CuO. Based on the EXAFS fitting curves of Sn_1_/CuO (supplementary Fig. S12B and C) and 0.1Zn_1_-Sn_1_/CuO (Fig. 2E and F) as well as the fitting parameters shown in Table S2 in the online supplementary material, the best-fitting result is that the isolated Sn atoms are coordinated with four O atoms with the mean bonding length of 1.95 Å. Further, X-ray photoelectron spectroscopy (XPS) measurements (Fig. S13 in the online supplementary material) show that the binding energy of the Cu 2p_3/2_ peak in CuO and Sn_1_/CuO is located at 933.48 eV, which corresponds to Cu^2+^ in CuO [31], in good agreement with the above XAS results. After incorporation with Zn atoms, the binding energy of Cu 2p_3/2_ peak shifts to the lower-energy side in comparison with that of CuO, and this shift is observed obviously in 0.1Zn_1_-Sn_1_/CuO, indicating an increase of the electron density on the Cu atoms with the coexistence of Sn and Zn atoms [32,33]. It evidences that there is an interaction between Sn and Zn atoms, and when the Zn content is 0.1 wt% relative to CuO, the interaction is the strongest. In spite of that, the spectra of Sn_1_/CuO and *x*Zn_1_-Sn_1_/CuO exhibit Sn 3d_5/2_ peaks with similar binding energies at 486.5 eV, which could be deconvoluted into two peaks at 486.3 eV and 487.3 eV, corresponding to Sn^2+^ and Sn^4+^, respectively (Figs S14–S17 in the online supplementary material). Besides, the peak of Zn 2p_3/2_ at about 1021.2 eV belongs to Zn^2+^ (Fig. S18 in the online supplementary material) [34]. The above results demonstrate the successful synthesis of a CuO catalyst with both Sn and Zn single atoms as co-promoters, and there generates strongly cooperative interaction between single-site Sn and Zn, which leads to the change in the electronic structure of the CuO catalyst.

### Catalytic synthesis of dimethyldichlorosilane via the Rochow reaction

**Figure 3.**
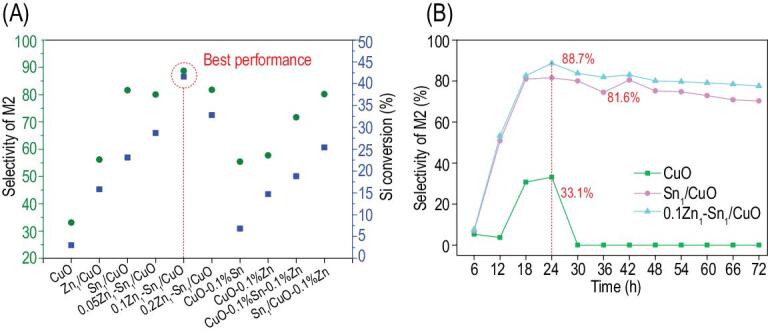
(A) The comparison of M2 selectivity and Si conversion in all samples. (B) The M2 selectivity as a function of time for CuO, Sn_1_/CuO and 0.1Zn_1_-Sn_1_/CuO in reaction of 72 h.

A vital industrialized reaction (Scheme S1 in the online supplementary material), the Rochow reaction, was used to evaluate the catalytic properties of *x*Zn_1_-Sn_1_/CuO and Sn_1_/CuO. This reaction generates methylchlorosilanes as the main products, such as methyltrichlorosilane (MeSiCl_3_, M1), dimethyldichlorosilane (Me_2_SiCl_2_, M2), trimethylchlorosilane (Me_3_SiCl, M3), together with trace amounts of other Si-containing compounds. Among them, M2 is the most desired as it is the most important monomer used for the synthesis of the organosilicon polymers. Although many Cu-based catalysts have been reported to date [35–37], there is still room to improve the M2 selectivity and yield. It is expected that Zn_1_-Sn_1_/CuO with its unique structure might exhibit an outstanding catalytic property. For comparison purposes, the four physical mixtures were also obtained, including (1) commercial Sn nanoparticles (Fig. S19A in the online supplementary material) and the synthesized CuO at the weight ratio of 1:1000 (denoted as CuO-0.1%Sn), (2) commercial Zn nanoparticles (supplementary Fig. S19B) and the synthesized CuO at the weight ratio of 1:1000 (denoted as CuO-0.1%Zn), (3) commercial Sn nanoparticles, commercial Zn nanoparticles and the synthesized CuO at the weight ratio of 1:1:1000 (denoted as CuO-0.1%Sn-0.1%Zn), and (4) commercial Zn nanoparticles and the synthesized Sn_1_/CuO at the weight ratio of 1:1000 (denoted as Sn_1_/CuO-0.1%Zn). Moreover, the sample of Zn_1_/CuO with the Zn species in the form of a single atom was also synthesized (Figs S20–S22 in the online supplementary material). Table 1 summarizes the reaction results at 325°C for 24 h under atmospheric pressure. As indicated, pure CuO (entry 1) gives an extremely low Si conversion and M2 selectivity, only 3% and 33.1%, respectively. Zn_1_/CuO (entry 2) shows improved Si conversion (15.8%) and M2 selectivity (56.2%); however, Sn_1_/CuO (entry 3) exhibits a remarkable increase in catalytic performance, both values reaching 23.2% and 81.6%, respectively. After introducing Zn into Sn_1_/CuO, a typical trend of the volcanic curve is observed for catalyst performance with increasing Zn content (entries 4–6). Notably, for the optimized 0.1Zn_1_-Sn_1_/CuO, Si conversion and M2 selectivity are increased to 41.6% and 88.7% (entry 5), 13.8 and 2.7 times higher than those of CuO, respectively. It is noticed that the catalysts of CuO-0.1%Sn, CuO-0.1%Zn, CuO-0.1%Sn-0.1%Zn and Sn_1_/CuO-0.1%Zn also display a certain degree of improvement in catalytic performance compared with pure CuO (entries 7–10). However, these values are still much lower than those of 0.1Zn_1_-Sn_1_/CuO (Fig. 3A), even using a large amount of commercial Sn and Zn nanoparticles (5 wt% of CuO, see Fig. S23 and Table S3 in the online supplementary material), suggesting that 0.1Zn_1_-Sn_1_/CuO possesses an incredibly high activity for this reaction. Moreover, compared with CuO and Sn_1_/CuO (Tables S4–S6 in the online supplementary material, Fig. 3B, and Fig. S24 in the online supplementary material), 0.1Zn_1_-Sn_1_/CuO exhibits a faster Si consumption rate and more stable M2 selectivity in the 72 h reaction on stream. These results indicate that the 0.1Zn_1_-Sn_1_/CuO catalyst possesses higher catalytic activity, selectivity and stability toward M2 than the other catalysts. As analyzed in the following section, this much-enhanced catalytic performance is associated with the cooperative interactions between the co-doped atomically dispersed promoters (Zn and Sn) and CuO, as well as the anchoring effect caused by defects.

**Table 1.** Catalytic performance of all samples.

		Product selectivity (%)^a^							
Entry	Catalyst	M2	M1	M3	M2H	M1H	LB	HB	*C*_Si_ (%)^b^
1	CuO	33.1	19.5	1.9	3.8	0.6	13.9	27.2	3.0
2	Zn_1_/CuO	56.2	22.0	4.0	0	5.7	11.8	0.3	15.8
3	Sn_1_/CuO	81.6	9.7	3.5	1.9	1.9	0.4	1.0	23.2
4	0.05Zn_1_-Sn_1_/CuO	80.0	13.8	2.5	1.2	2.2	0.1	0.2	28.7
5	0.1Zn_1_-Sn_1_/CuO	88.7	5.2	2.8	1.5	0.1	1.2	0.5	41.6
6	0.2Zn_1_-Sn_1_/CuO	81.7	8.4	4.2	1.8	2.8	0.4	0.7	32.8
7	CuO-0.1%Sn	55.4	28.1	1.7	2.9	0.5	6.8	4.6	6.8
8	CuO-0.1%Zn	57.7	19.6	6.7	1.0	4.2	7.4	3.4	14.7
9	CuO-0.1%Sn-0.1%Zn	71.7	14.8	3.4	2.8	2.3	4.1	0.9	18.8
10	Sn_1_/CuO-0.1%Zn	80.2	11.9	4.5	1.4	0.7	0	1.3	25.4

Reaction conditions: 325°C, 24 h, 10 g Si, 0.5 g catalyst, 25 mL min^−1^ MeCl gas. ^a^M2, dimethyldichlorosilane (Me_2_SiCl_2_); M1, methyltrichlorosilane (MeSiCl_3_); M3, trimethylchlorosilane (Me_3_SiCl); M2H, dimethylchlorosilane (Me_2_SiHCl); M1H, methyldichlorosilane (MeSiHCl_2_); LB, low boiler compounds; HB, high boiler compounds. ^b^Conversion of Si.

The waste contact masses (unreacted residue) after a 24 h reaction with the highest selectivity toward M2 for all the catalysts were characterized. From XRD analysis (Fig. S25 in the online supplementary material), it is found that the Cu*_x_*Si species, which is considered to be the real catalytic active phase [38,39], was formed and its strongest peak intensity was obtained on 0.1Zn_1_-Sn_1_/CuO, showing that 0.1Zn_1_-Sn_1_/CuO has the strongest ability to generate the active Cu*_x_*Si species. Scanning electron microscopy (SEM) observation (Fig. S26 in the online supplementary material) confirms that the shapes of the Zn_1_-Sn_1_/CuO catalyst remained unchanged, and there was an occurrence of Si etching during the catalytic process. Among all the catalysts, the extent of the Si etching was most severe on 0.1Zn_1_-Sn_1_/CuO. These results confirm that through controlling the surface structure of 0.1Zn_1_-Sn_1_/CuO, the formation of the Cu*_x_*Si active phase can be enhanced.

### Density functional theory calculation

The DFT calculations support that Sn and Zn dopants are dispersed atomically on the CuO(110) surfaces. The calculated results show that for two Sn atoms to substitute two Cu atoms on CuO(110), the tendency is for substitution to occur at two Cu sites that are far from each other. Compared to the system of substituting two nearby Cu atoms, the energy for substituting two far-away Cu atoms is 0.14 eV lower (Fig. S27A in the online supplementary material). We also find that Sn energetically prefers to occupy a surface Cu site rather than a bulk Cu site. The energy difference between the surface and bulk sites is −1.76 eV/atom (*E*_surface_ = −459.28 eV while *E*_bulk_ = −457.52 eV).

Similarly, Zn atoms also prefer to separate from each other, but the energy difference of 0.08 eV for replacing two Cu atom by Zn atoms is smaller than that of the Sn doping case (supplementary Fig. S27B). We also find that Zn tends to occupy a Cu site that locates next to Sn if Sn exists on the surface, namely Sn and Zn atoms prefer to form pairs on CuO(110). The calculated result shows that forming a Sn-Zn pair is energetically 0.06 eV lower than separating Sn and Zn far away (supplementary Fig. S27C). Thus Zn prefers to fill in the nearby Cu vacancies caused by Sn doping. These theoretical results are consistent with the experimental observations.

**Figure 4.**
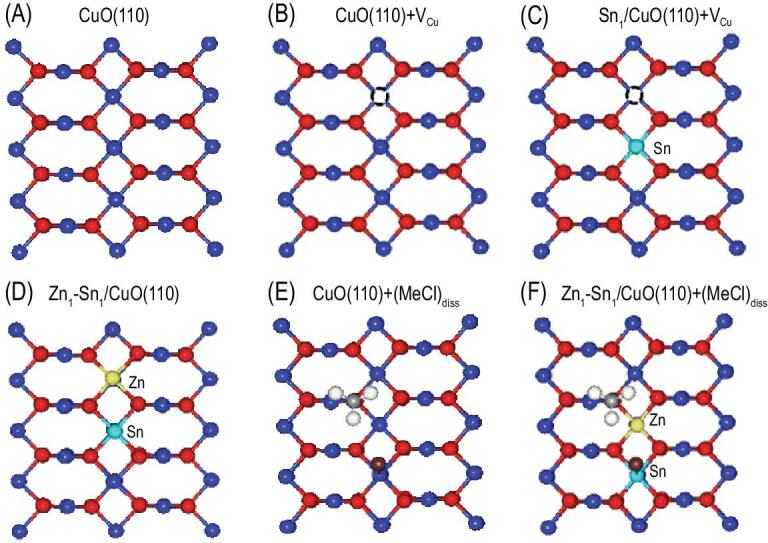
Optimized surface atomic structures without and with MeCl adsorption. (A) CuO(110); (B) CuO(110) with a Cu vacancy; (C) Sn-doped CuO(110) with a Cu vacancy; (D) Zn occupying the Cu vacancy to form a Sn-Zn pair; (E) MeCl dissociative adsorption on undoped CuO(110); (F) MeCl dissociative adsorption on Sn-Zn pair doped CuO(110). Color scheme: O, red; Cu, blue; C, gray; H, white; Cl, brown; Sn, bright blue; Zn, yellow.

Figure 4A shows the atomic structure of CuO(110). We calculated the formation energies of a Cu vacancy for a clean CuO(110) surface (Fig. 4B), an Sn-doped CuO(110) surface (Fig. 4C) and a Zn-doped CuO(110) surface (Fig. S28 in the online supplementary material). It is found that on the Sn-doped CuO(110) surface, the formation energy is 0.78 eV lower than that on the clean CuO(110), while on the Zn-doped CuO(110) surface, it is 0.13 eV higher than that on the clean CuO(110). These results confirm that it is easier to form Cu vacancies in the Sn-doped case. The Sn doping facilitates the formation of Cu vacancies on CuO, and the latter can anchor Zn and achieve its single-atomic dispersion (Fig. 4D).

According to the previous reports [38,39], the catalytic reaction involves two stages: one is the transformation of catalyst into the Cu*_x_*Si active phase, and the other is the adsorption and activation of gaseous MeCl on the Cu*_x_*Si active phase to form gaseous products. Our previous work [40] has demonstrated that the rapid generation of free Cu atoms should be the crucial step to form Cu*_x_*Si, which is closely related to the dissociative adsorption strength of MeCl. Therefore, the dissociative adsorption behaviors of MeCl on the clean CuO(110) (Fig. 4E), Sn-doped CuO(110) (Fig. S29 in the online supplementary material), Zn-doped CuO(110) (Fig. S30 in the online supplementary material) and Sn-Zn pair-doped CuO(110) (Fig. 4F) were further investigated by DFT calculations. CH_3_ tends to adsorb on the O top site, and the adsorption energy is only slightly affected by doping, while the strongest adsorption occurs for Sn doping (Table S7 in the online supplementary material). However, we found a significant energy difference for Cl adsorption, and the most substantial adsorption occurs on Sn-doped CuO(110). Interestingly, the dissociative adsorption of MeCl is energetically unfavorable for clean Cu(110), but turns out to be energetically favorable upon doping, especially in the case of Sn-Zn pair and Sn-doping. Such an enhancement effect will facilitate the formation of copper chloride species and Cu atoms subsequently. As a result, the diffusion of Cu atoms to the surface of the Si matrix is enhanced, and thus the formation of the Cu*_x_*Si active phase promoted.

The electron transfers for CuO(110) with Sn or Zn doping and Sn-Zn pair doping are calculated as well, and the results are summarized in Table S8 in the online supplementary material. As discussed above, the doped Sn atoms can help to form surface Cu vacancies and make it possible to form Sn-Zn pairs on the surface. The results indicate that for Sn and Sn-Zn pair doped cases, the oxygen atoms bonded with Sn and Zn gain more electrons than those in clean CuO(110). In turn, the oxygen atoms gain fewer electrons from Cu atoms. Especially in the Sn-Zn pair doped case, Cu atoms transfer the fewest electrons to oxygen atoms, which means that Cu atoms have more valence electrons compared to the undoped CuO(110) surface. These theoretical results are consistent with the XPS measurements that electron density of the Cu atoms becomes higher for 0.1Zn_1_-Sn_1_/CuO relative to Sn-doped and clean CuO(110). Both experimental and theoretical results indicate that Sn and Zn interact with each other and have cooperative effects on the catalytic performance. Therefore, we can understand why Zn_1_-Sn_1_/CuO exhibits the best performance among various measured catalysts.

## CONCLUSION

In summary, a novel CuO catalyst with atomically dispersed Sn and Zn as co-promoters has been successfully prepared via a facile hydrothermal method followed by wet impregnation. In the synthesis, single-site Sn is first incorporated into the lattice of CuO catalysts during hydrothermal treatment to generate a large number of surface Cu vacancies, which can then be used to anchor Zn atoms. This novel catalyst is highly active and stable towards M2 synthesis in the Rochow reaction. DFT calculation further confirms that the single-site Sn facilitates the generation of Cu vacancies, which can capture Zn and realize a single-atomic dispersion of Zn. The synergistic interaction between single-site Sn and Zn leads to the change in the electronic structure of the CuO catalyst, which promotes the adsorption of reactant MeCl and formation of the Cu*_x_*Si active phase, thereby leading to the enhanced catalytic performance. This work provides a new understanding of the synergistic effect among various promoters and will offer avenues to the design of new co-promoters in catalysts for industrial reactions.

## METHODS

### Reagents and chemicals

All the chemicals were of analytical grade and used without further purification. These chemicals include stannic chloride pentahydrate (SnCl_4_·5H_2_O, 99.0%, Xilong Chemical Co., Ltd), sodium hydroxide (NaOH, 96.0%, Xilong Chemical Co., Ltd), copper sulfate pentahydrate (CuSO_4_·5H_2_O, 99.0%, Xilong Chemical Co., Ltd), ethanol (CH_3_CH_2_OH, 99.0%, Beijing Chemical Reagent Co., Ltd), toluene (C_7_H_8_, 99.0%, Beijing Chemical Reagent Co., Ltd) zinc dichloride (ZnCl_2_, 98.0%, Fuchen Chemical Reagent Factory), Sn powder (99.9%, Sinopharm Chemical Reagent Co., Ltd) and Zn powder (99.9%, Sinopharm Chemical Reagent Co., Ltd).

### Materials preparation

#### Synthesis of Sn_1_/CuO, Zn_1_/CuO and CuO

Sn_1_/CuO was synthesized by a reported method with some modifications [24]. First, 24.96 g (0.1 mol) of CuSO_4_·5H_2_O and 0.0186 g (5.32 × 10^−2^ mmol) of SnCl_4_·5H_2_O were dissolved in 100 mL of deionized water under vigorous stirring and in an ice-water bath to form a homogeneous blue solution. Then, 200 mL of NaOH solution (1.2 mol/L) was slowly added, and the mixture was continuously stirred for 15 min. After being refrigerated (3°C) for 24 h, the mixture was sealed and maintained at 130°C for 18 h. Finally, the product was filtered after cooling, washed with distilled water and ethanol several times, and dried at 60°C for 8 h. The synthesis of Zn_1_/CuO followed with the same procedures except that SnCl_4_·5H_2_O was replaced by ZnCl_2_. Similarly, the CuO sample was also synthesized following the same procedures but without adding SnCl_4_·5H_2_O.

#### Synthesis of *x*Zn_1_-Sn_1_/CuO

The *x*Zn_1_-Sn_1_/CuO samples were prepared by using the impregnated method, where *x* refers to the weight ratio of zinc to CuO. First, 4.00 g of Sn_1_/CuO was added in 100 mL of ethanol to obtain suspension A. A desirable amount of ZnCl_2_ (0.0042 g, 0.0084 g and 0.0168 g, corresponding to 0.05 wt%, 0.1 wt% and 0.2 wt% relative to CuO, respectively) was dissolved in 20 mL of distilled water to obtain solution B. Subsequently, solution B was slowly added into the suspension A under stirring and the obtained mixture was continuously stirred for 150 min. The product was filtered, washed with distilled water and ethanol several times, and dried at 60°C overnight under vacuum. Finally, the resulting powder was calcined at 400°C for 3 h to obtain *x*Zn_1_-Sn_1_/CuO.

#### Catalytic tests

The catalyst test was performed using a fixed-bed reactor. A 10.00 g amount of silicon powder (150 mesh, provided by Tangshan Sanyou Group Co. Ltd) was mixed homogeneously with 0.50 g of the prepared catalyst to form the contact mass, which was then loaded into the glass reactor. The reactor system was initially purged by methyl chloride (CH_3_Cl or MeCl, Zhejiang Guoya Gas Co., Ltd) at 20°C for 0.5 h. Afterwards, the temperature was raised to 325°C (5°C min^−1^) for reaction for 24 h. The flow rate of MeCl was 25 mL min^−1^. The gas product was cooled into liquid phase with a circulator bath controlled at −20°C by a programmable thermal circulator (GDH series, Ningbo Xinzhi Biological Technology Co., Ltd). Gas chromatography (Agilent Technologies GC-7890A, KB-201 capillary column (60 m), thermal conducting detector (TCD)) was used to quantitatively analyze the products, which were mainly composed of methyltrichlorosilane (MeSiCl_3_, M1), dimethyldichlorosilane (Me_2_SiCl_2_, M2), trimethylchlorosilane (Me_3_SiCl, M3), methyldichlorosilane (MeSiHCl_2_, M1H), dimethylchlorosilane (Me_2_SiHCl, M2H), low boiler compounds (LB) and high boiler compounds (HB). The Si conversion and M2 selectivity are calculated as follows:

(1) \begin{equation*}{\rm{Si\, conversion\!\!:\, }}{C_{{\rm{Si}}}}{\rm{(\% ) }}\! =\! {\rm{ }}\frac{{{m_{{\rm{Si, before}}}} - {m_{{\rm{Si, after}}}}}}{{{m_{{\rm{Si, before}}}}}} \times {\rm{100 }}\end{equation*}

(2) \begin{equation*}{\rm{M2\, selectivity\!\!:\, }}S_{\rm{M2}}{\rm{(\% )}} = {\rm{ }}\frac{{{m_{{\rm{M2}}}}}}{{\sum\nolimits_{{\rm{i = }}1}^3 m_ {M{\rm{i}}}}} \times {\rm{100 }}\end{equation*}

Here, *m*_Si, before_ and *m*_Si, after_ in Formula (1) represent the mass of Si powder before and after the reaction, respectively, and *m* in Formula (2) is the mass of the products (as a percentage; peak area calibrated with response factor).

#### Characterization

XRD analysis was performed on a PANalytica X’Pert PRO MPD using CuKα radiation (*k* = 1.5418 Å) at 40 kV and 40 mA. The size and shape of the as-prepared samples were observed using a cold field-emission SEM (SU8020, HITACHI, Japan) and a field-emission TEM (Tecnai G^2^ F20 S-TWIN, FEI, USA), and a HAADF-STEM (JEM-ARM200F, JEOL, Japan) operated at 200 kV. The contents of Zn and Sn were determined by ICP-OES (Optima 5300DV, Perkin Elmer, USA) analysis. XPS spectra (Model VG ESCALAB 250 spectrometer, Thermo Electron, UK) were recorded using an AlKα X-ray source (hm = 1486.6 eV) radiation to analyze the surface chemical composition of samples. This reference gave BE values with an error within ±0.1 eV. Atomic force microscopy (AFM) (MultiMode 8, BRUKER, Germany) was used to observe the relative roughness of the sample surface.

The X-ray absorption fine structure (XAFS) spectra data (Cu K-edge) were collected at 1W1B station in Beijing Synchrotron Radiation Facility (BSRF, operated at 2.5 GeV with a maximum current of 250 mA). The data were collected in transmission mode. All samples were pelletized as disks of 13 mm diameter with 1 mm thickness using graphite powder as a binder. The acquired EXAFS data were processed according to the standard procedures using the ATHENA and ARTEMIS implemented in the IFEFFIT software packages. The fitting detail is described below.

The EXAFS spectra were obtained by subtracting the post-edge background from the overall absorption and then being normalized with respect to the edge-jump step. Subsequently, the χ(*k*) data of Fourier were transformed to real (*R*) space using a hinging window (d*k* = 1.0 Å^−1^) to separate the EXAFS contributions from different coordination shells. To obtain the quantitative structural parameters around central atoms, least-squares curve parameter fitting was performed using the ARTEMIS module of IFEFFIT software packages.

The following EXAFS equation is used:

$$\begin{eqnarray*}\chi (k) &=& \sum\limits_j {\frac{{{N_j}S_o^2{F_j}(k)}}{{kR_j^2}}} \exp [ - 2{k^2}\sigma _j^2]\nonumber\\
&&\times\exp \left[ {\frac{{ - 2{R_j}}}{{\lambda (k)}}} \right]\sin [2k{R_j} + {\phi _j}(k)]\end{eqnarray*}$$

*S*_0_^2^ is the amplitude reduction factor, *F_j_*(*k*) is the effective curved-wave backscattering amplitude, *N_j_* is the number of neighbors in the *j*th atomic shell, *R_j_* is the distance between the X-ray absorbing central atom and the atoms in the *j*th atomic shell (backscatterer), *λ* is the mean free path in Å, *φ_j_*(*k*) is the phase shift (including the phase shift for each shell and the total central atom phase shift), *σ_j_* is the Debye–Waller parameter of the *j*th atomic shell (variation of distances around the average *R_j_*). The functions *F_j_*(*k*), *λ* and *φ_j_(k)* are calculated with the *ab initio* code FEFF8.2. The coordination numbers of model samples are fixed as the nominal values. The obtained *S*_0_^2^ is fixed in the subsequent fitting, while the internal atomic distances *R*, Debye–Waller factor *σ^2^*, and the edge-energy shift Δ*E*_0_ are allowed to run freely.

#### Computational methods

All calculations were performed within the framework of DFT as implemented in the Vienna Ab initio Simulation Package (VASP) code [41–43]. The electron–ion interaction is described using the projector augmented wave method [44,45]. We employ the generalized gradient approximation (GGA) in the Perdew–Burke–Ernzerhof (PBE) form for the exchange–correlation functional [46]. The CuO(110) surface is simulated by a slab model constructed with the theoretical equilibrium lattice constants. The vacuum thickness is 15 Å. The atoms in the top four layers are allowed to relax until the forces on those atoms are less than 0.02 eV Å^−1^, while the atoms in the bottom two layers are fixed at the bulk lattice sites. The energy cutoff for the plane wave basis is 500 eV for all our calculations. The Brillouin zone integration is sampled with the 4 × 4 × 1 k-point mesh by the Monkhorst–Pack scheme [47].

## Supplementary Material

nwz196_Supplemental_File
