# Supplementary material for: Single-atom Sn-Zn pairs in CuO catalyst promote dimethyldichlorosilane synthesis
Source: Natl Sci Rev. 2019 Nov 28;7(3):600–8. doi: 10.1093/nsr/nwz196 (PMC8288878; doi:10.1093/nsr/nwz196)
Supplement: nwz196_Supplemental_File [file nwz196_supplemental_file.docx]

**Supplementary Data**

**Single-atom Sn-Zn Pairs in CuO Catalyst Promote Dimethyldichlorosilane Synthesis**

Qi Shi,^a,b^ Yongjun Ji,^b,c,*^ Wenxin Chen,^d^ Yongxia Zhu,^b^ Jing Li,^b^ Hezhi Liu,^b^ Zhi Li,^e^ Shubo Tian,^e^ Ligen Wang,^a,*^ Ziyi Zhong,^f^ Limin Wang,^a,*^ Jianmin Ma,^g^ Yadong Li^e^ and Fabing Su^b,c,h,*^

*^a^ Gripm Advanced Materials Co., Ltd., Beijing, 101407, China*

*^b^ State Key Laboratory of Multiphase Complex Systems, Institute of Process Engineering, Chinese Academy of Sciences, Beijing 100190, China*

*^c^ Zhongke Langfang Institute of Process Engineering, Fenghua Road No 1, Langfang Economic & Technical Development Zone, Hebei 065001, China*

*^d^ Beijing Key Laboratory of Construction Tailorable Advanced Functional Materials and Green Applications, School of Materials Science and Engineering, Beijing Institute of Technology, Beijing 100081, China*

*^e^ Department of Chemistry, Tsinghua University, Beijing 100084, China*

*^f^ College of Engineering, Guangdong Technion Iarael Institute of Technology (GTIIT), 241 Daxue Road, Shantou 515063, China; Technion–Israel Institute of Technology (IIT), Haifa, 32 000, Israel*

*^g^ School of Physics and Electronics, Hunan University, Changsha 410082, China*

*^h^ Institute of Industrial Chemistry and Energy Technology, Shenyang University of Chemical Technology, Shenyang 110142, China*

*To whom correspondence should be addressed. E-mail address: [yjji@ipe.ac.cn](mailto:yjji@ipe.ac.cn) (Y. Ji); [lgwang16@126.com](mailto:lgwang16@126.com) (L. Wang); wang@gripm.com (L. Wang); fbsu@ipe.ac.cn (F. Su)

**Supplementary Figures and Tables**


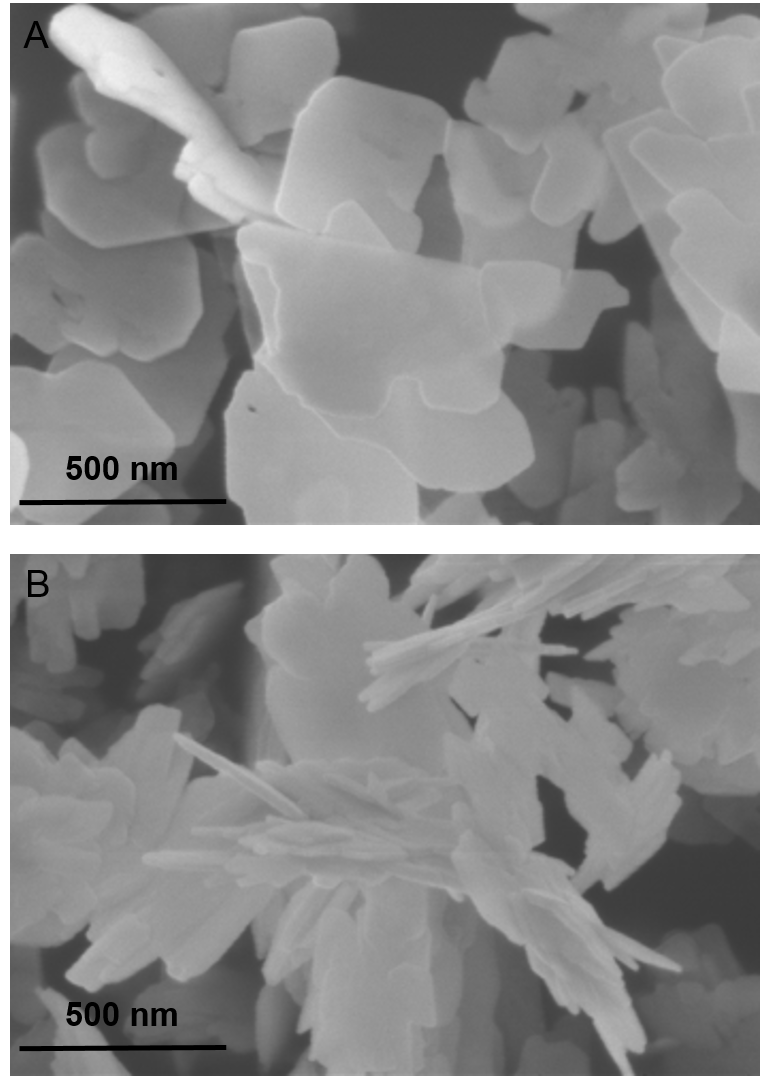


**Figure S1.** SEM images of (A) CuO and (B) Sn_1_/CuO.

**
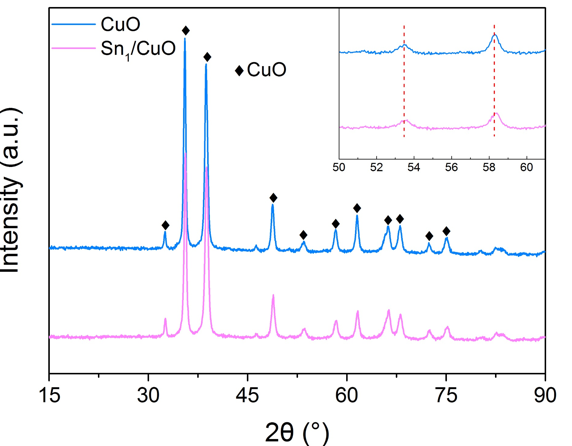
**

**Figure S2.** XRD patterns of CuO and Sn_1_/CuO. The insert is enlarged view in the 2*θ* angle range of 50–61°.


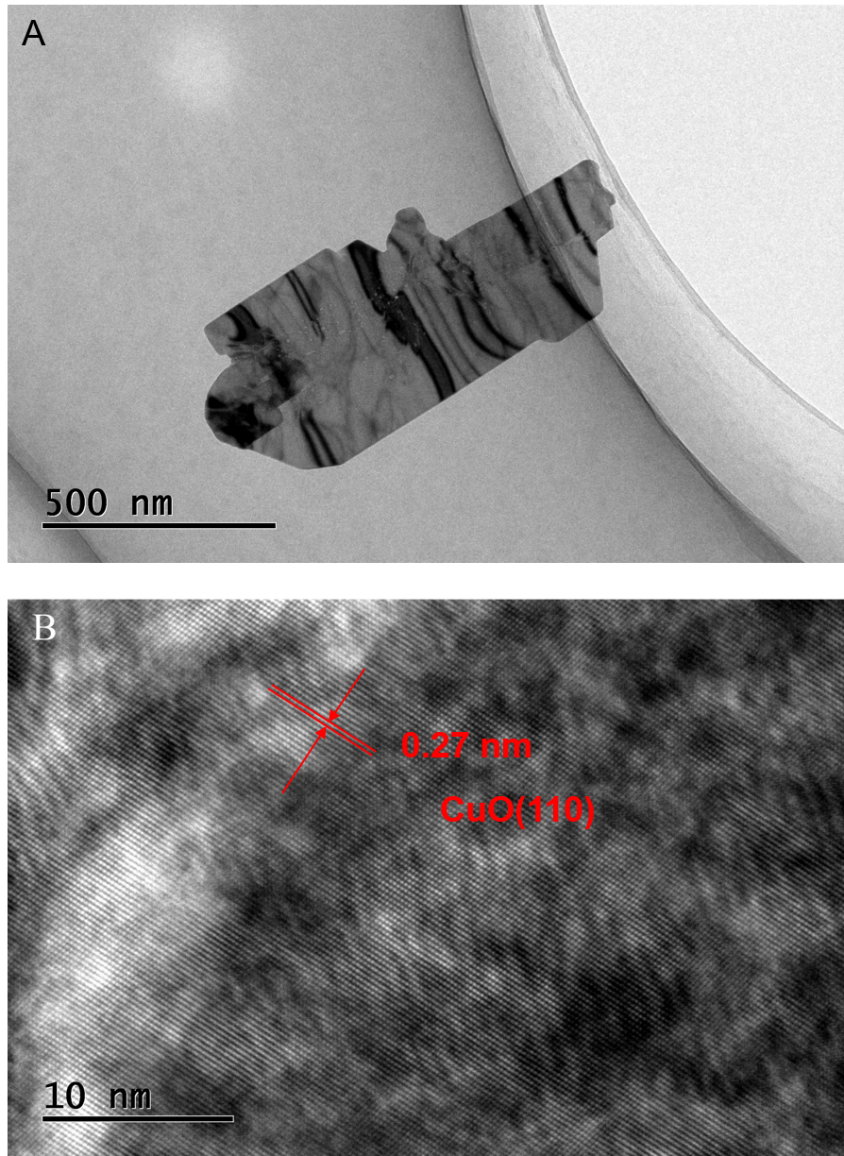


**Figure S3.** (A) TEM and (B) HRTEM images of CuO.


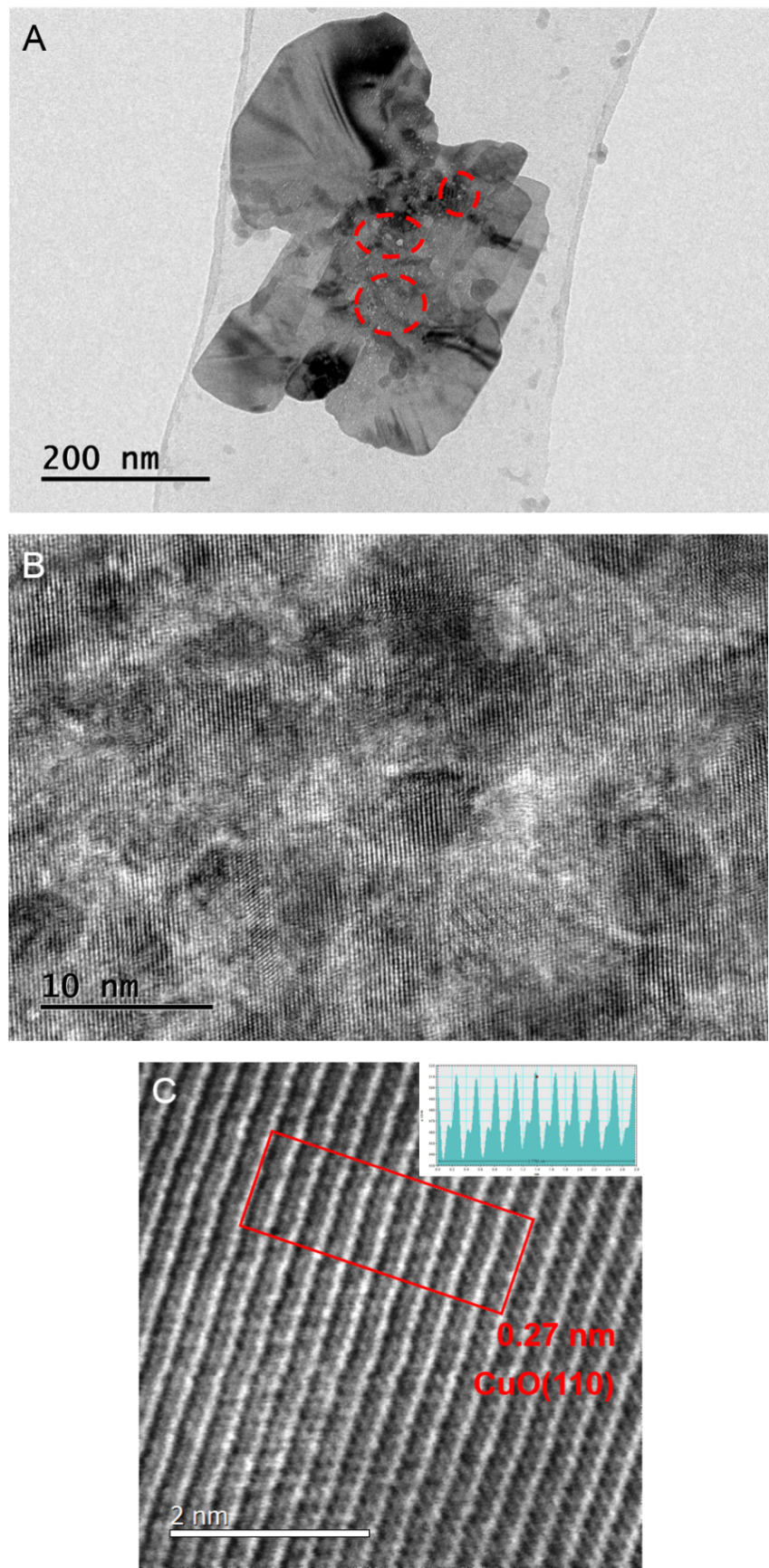


**Figure S4.** (A) TEM, (B) HRTEM, and (C) HADDF-STEM images of Sn_1_/CuO. The red dotted circle represents the depression on the surface of the sample.


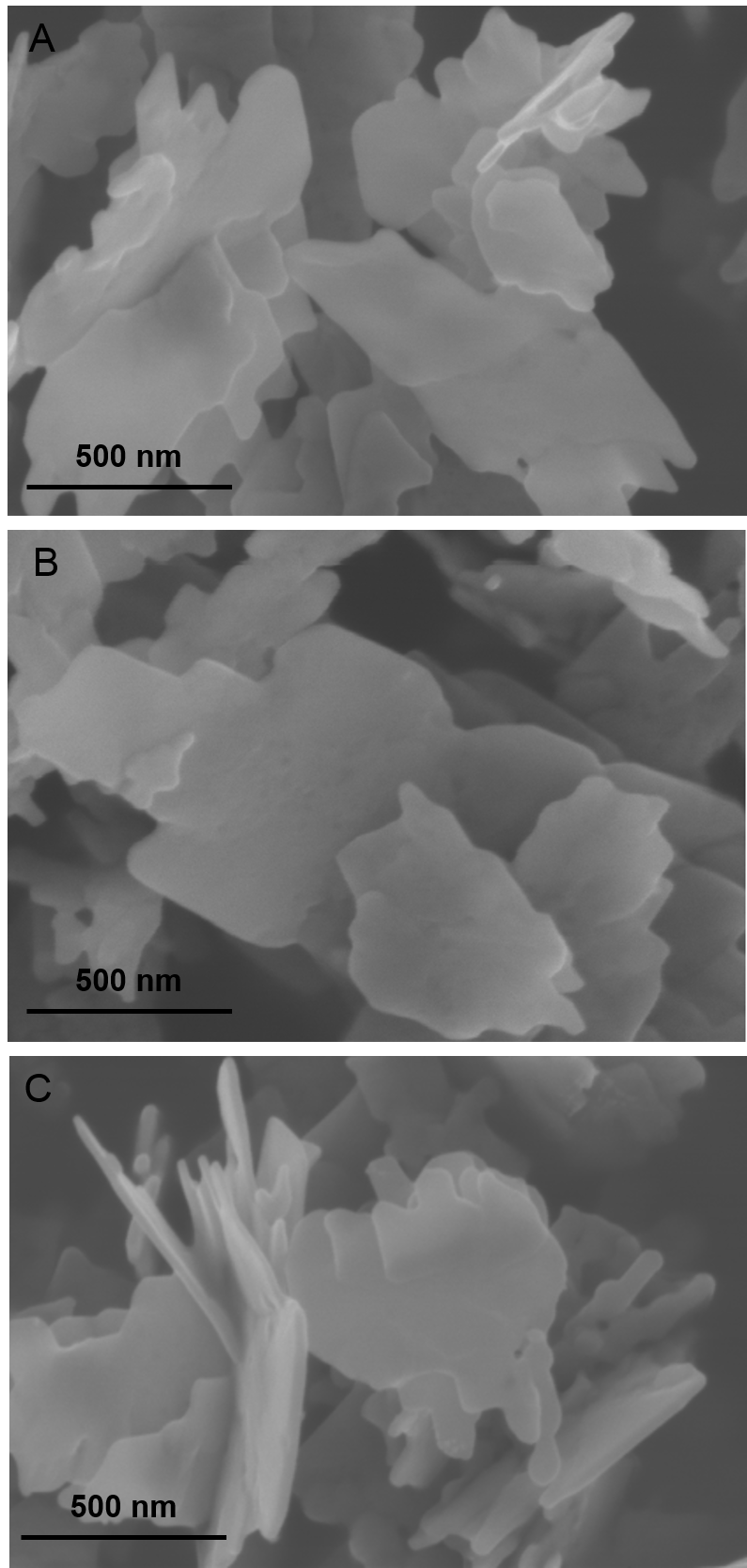


**Figure S5.** SEM images: (A) 0.05Zn_1_-Sn_1_/CuO, (B) 0.1Zn_1_-Sn_1_/CuO, and (C) 0.2Zn_1_-Sn_1_/CuO.

**
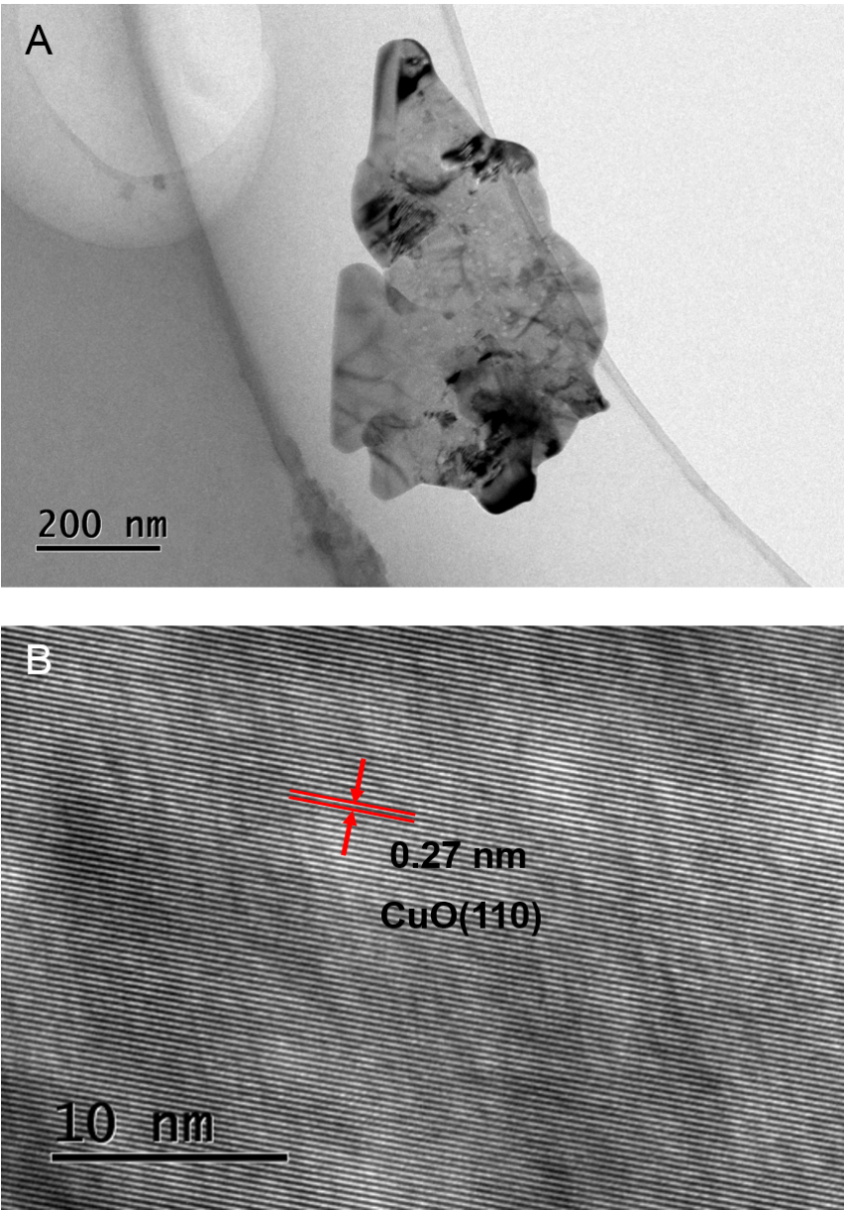
**

**Figure S6.** (A) TEM and (B) HRTEM images of 0.05Zn_1_-Sn_1_/CuO.

**
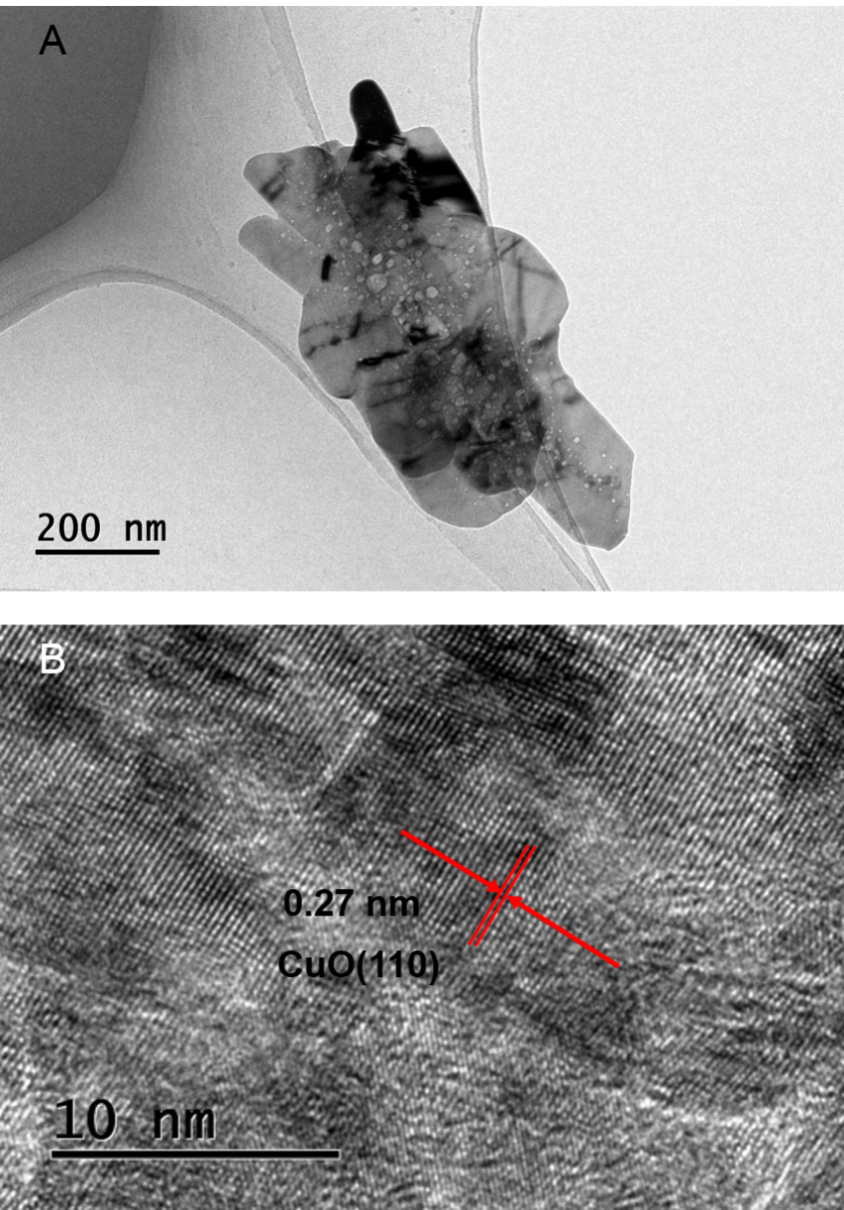
**

**Figure S7.** (A) TEM and (B) HRTEM images of 0.1Zn_1_-Sn_1_/CuO.


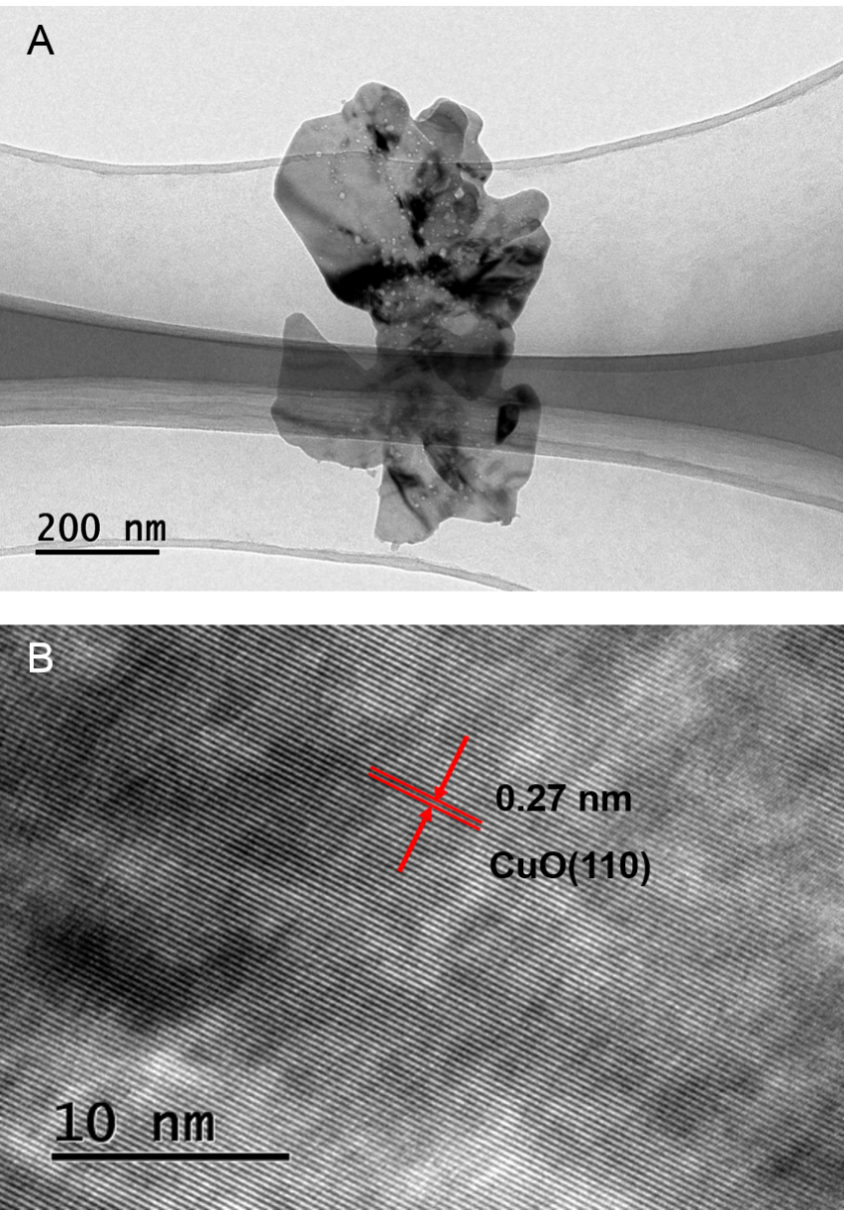


**Figure S8.** (A) TEM and (B) HRTEM images of 0.2Zn_1_-Sn_1_/CuO.

**
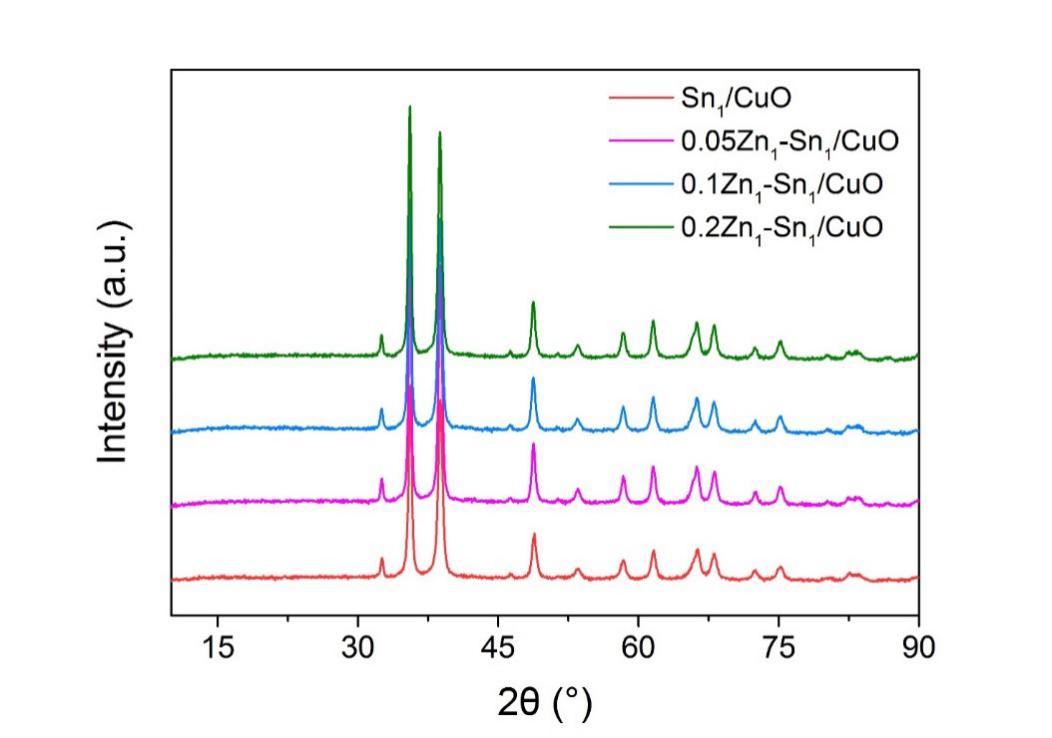
**

**Figure S9.** XRD patterns of Sn_1_/CuO, 0.05Zn_1_-Sn_1_/CuO, 0.1Zn_1_-Sn_1_/CuO and 0.2Zn_1_-Sn_1_/CuO.


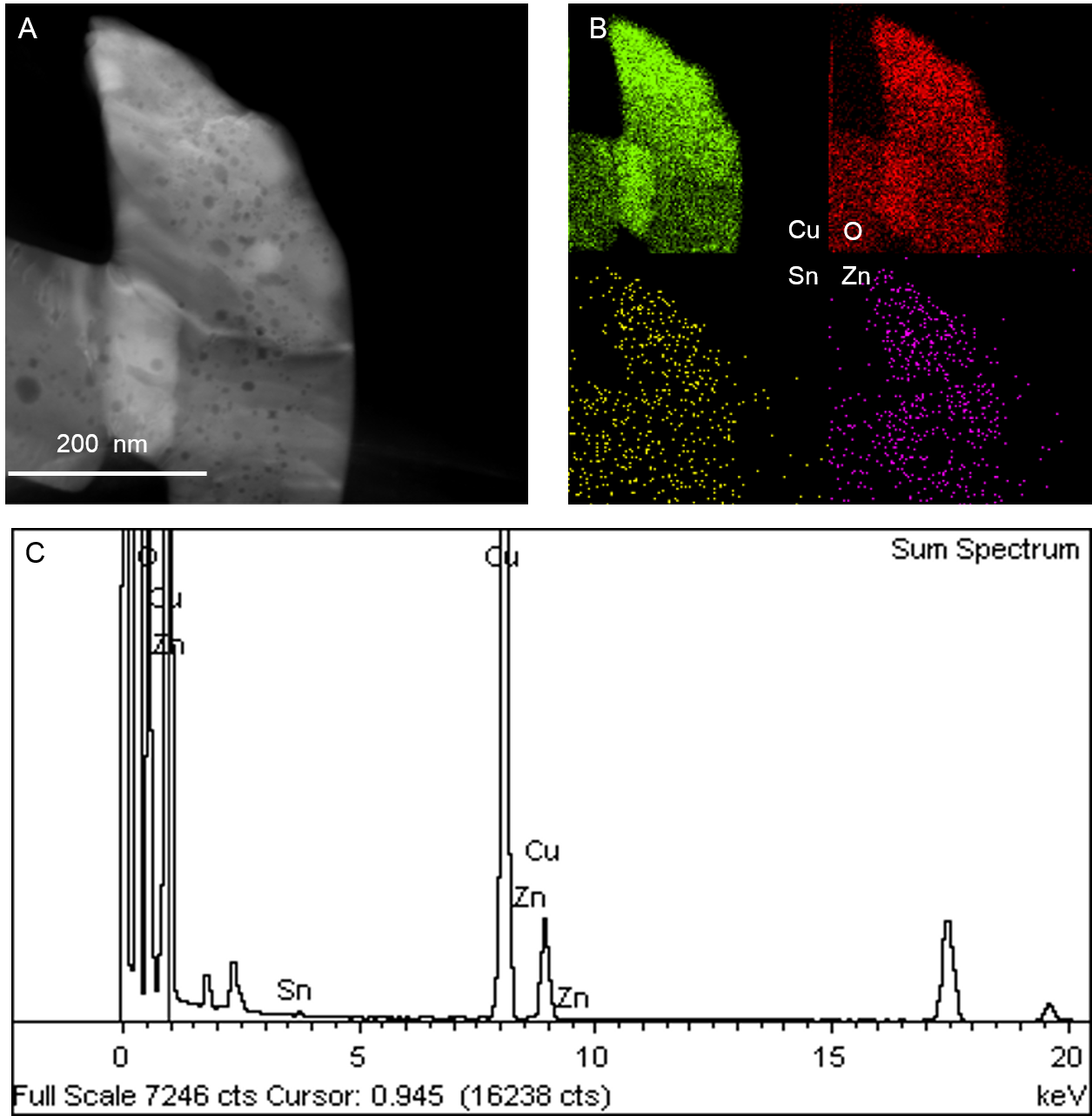


**Figure S10.** (A) HAADF-STEM image and (B) the corresponding EDS mappings as well as (C) the **EDX spectra** of 0.1Zn_1_-Sn_1_/CuO.

**Table S1.** The contents of Zn and Sn in various samples.

| Sample | Sn (wt%)^a^ | Sn (wt%)^b^ | Zn (wt%)^c^ | Zn (wt%)^d^ |
| --- | --- | --- | --- | --- |
| Sn_1_-CuO | 0.1 | 0.1 |  |  |
| 0.05Zn_1_-Sn_1_/CuO | 0.1 | 0.1 | 0.05 | 0.03 |
| 0.1Zn_1_-Sn_1_/CuO | 0.1 | 0.1 | 0.1 | 0.09 |
| 0.2Zn_1_-Sn_1_/CuO | 0.1 | 0.1 | 0.2 | 0.18 |

^a^ The theoretical weight ratio of Sn relative to CuO; ^b^ The actual weight ratio of Sn relative to CuO determined by ICP; ^c^ The theoretical weight ratio of Zn relative to CuO; ^d^ The actual weight ratio of Zn relative to CuO determined by ICP.

**
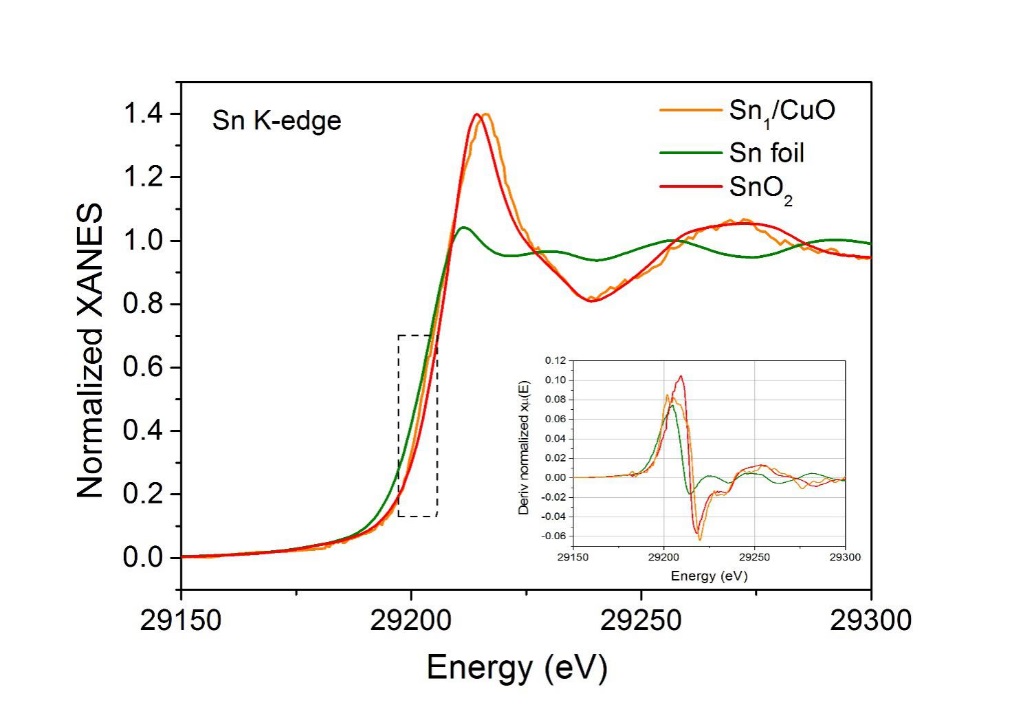
**

**Figure S11.** The normalized Sn K-edge XANES (inset is the deriv image) spectra.

**
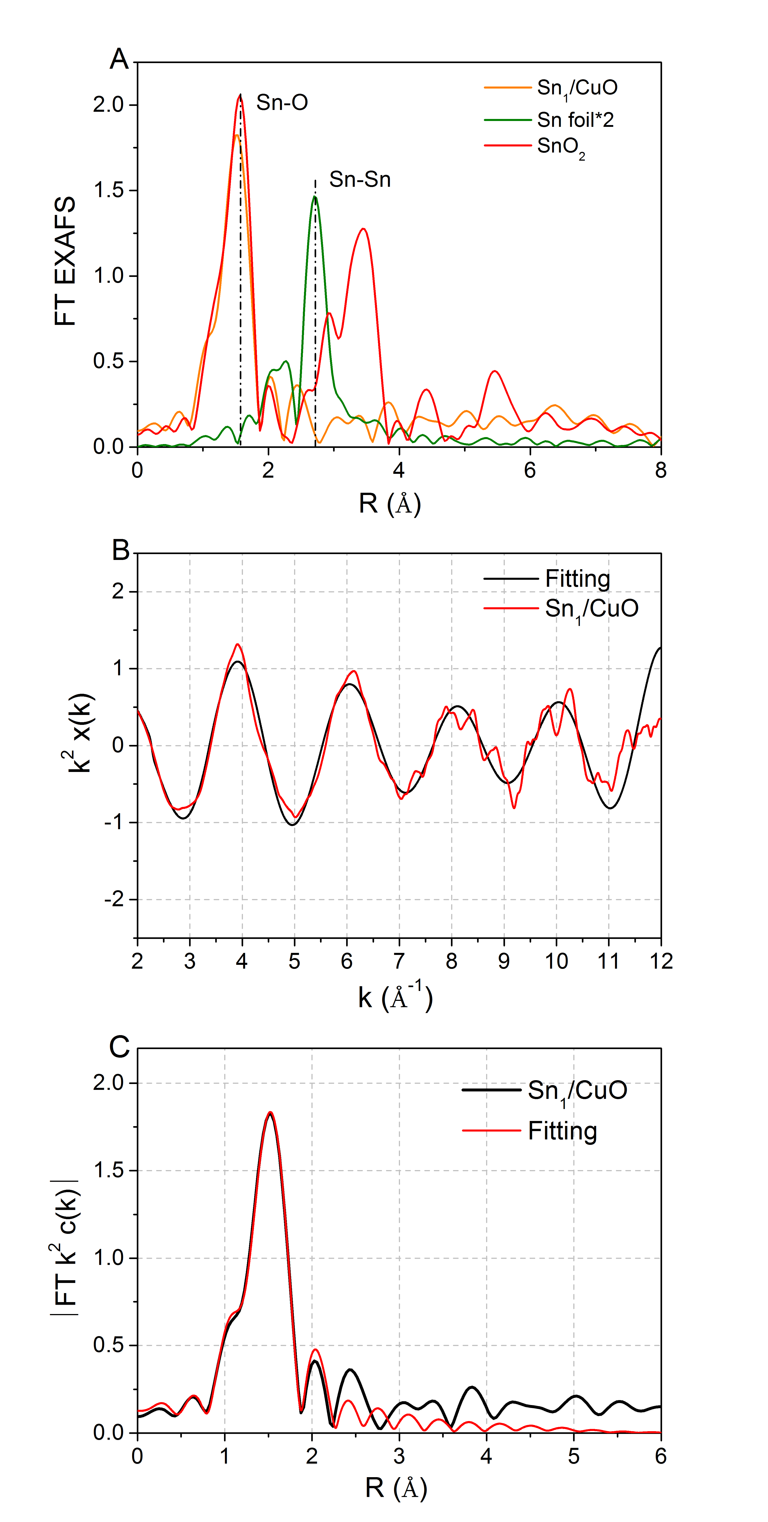
**

**Figure S12.** (A) FT k^2^-weighted EXAFS spectra of Sn, (B) k space EXAFS spectrum of the Sn_1_/CuO at the Sn K-edge and (C) the corresponding FT-EXAFS fitting curves of Sn_1_/CuO.

**Table S2.** Structural parameters extracted from the Sn K-edge EXAFS fitting. (S_0_^2^=0.85)

| **sample** | **Scattering pair** | **CN** | **R(Å)** | **σ^2^(10^-3^Å^2^)** | **ΔE_0_(eV)** | **R factor** |
| --- | --- | --- | --- | --- | --- | --- |
| Sn_1_/CuO | Sn-O | 3.8 | 1.95 | 4.5 | -2.0 | 0.005 |
| 0.1Zn_1_-Sn_1_/CuO | Sn-O | 4.3 | 1.95 | 5.6 | -1.5 | 0.006 |

S_0_^2^ is the amplitude reduction factor; CN is the coordination number; R is interatomic distance (the bond length between central atoms and surrounding coordination atoms); σ^2^ is Debye-Waller factor (a measure of thermal and static disorder in absorber-scatterer distances); ΔE_0_ is edge-energy shift (the difference between the zero kinetic energy value of the sample and that of the theoretical model). R factor is used to value the goodness of the fitting.

Error bounds that characterize the structural parameters obtained by EXAFS spectroscopy were estimated as N ± 20%; R ± 1%; σ^2^ ± 20%; ΔE_0_ ± 20%.


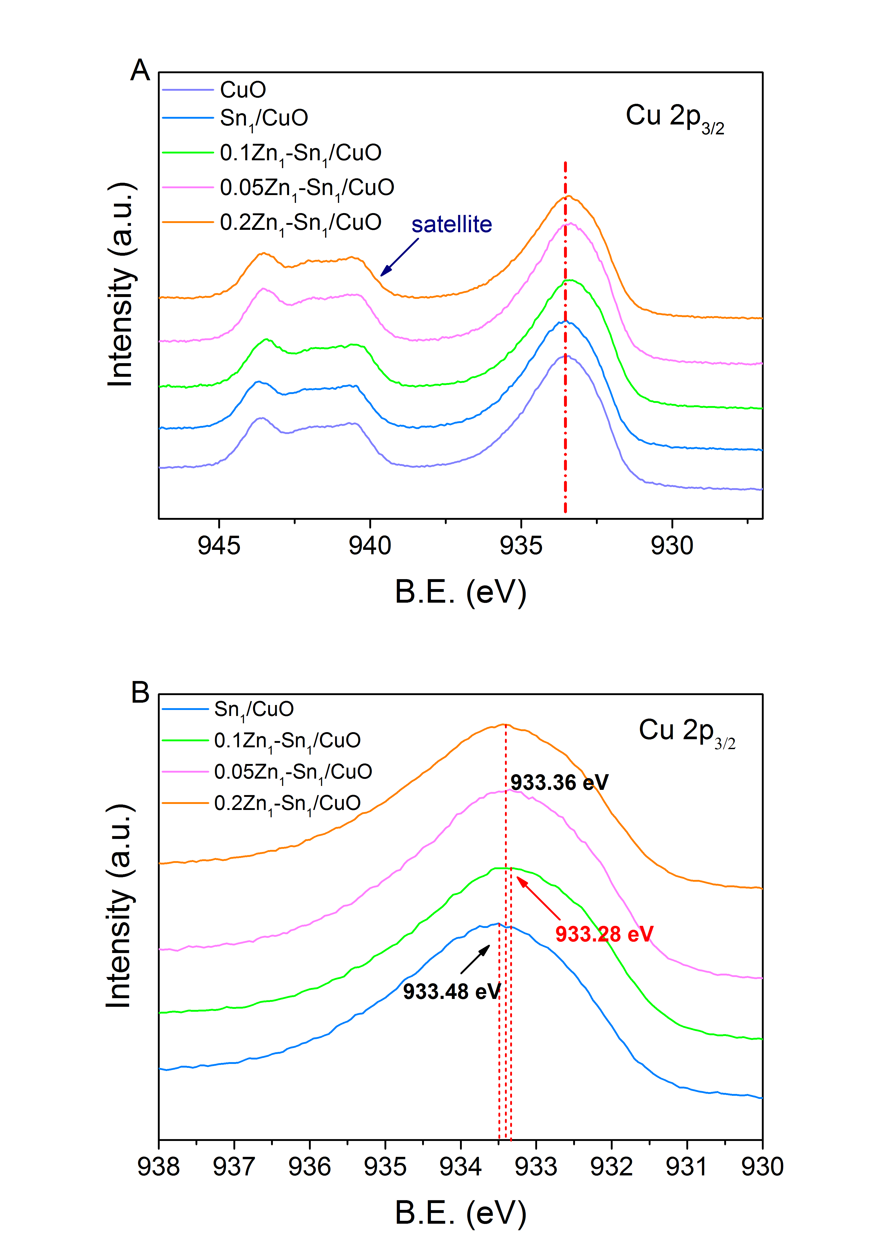


**Figure S13.** (A) XPS spectra and (B) the corresponding magnification of Cu 2p_3/2_ in CuO, Sn_1_/CuO, 0.05Zn_1_-Sn_1_/CuO, 0.1Zn_1_-Sn_1_/CuO, and 0.2Zn_1_-Sn_1_/CuO.


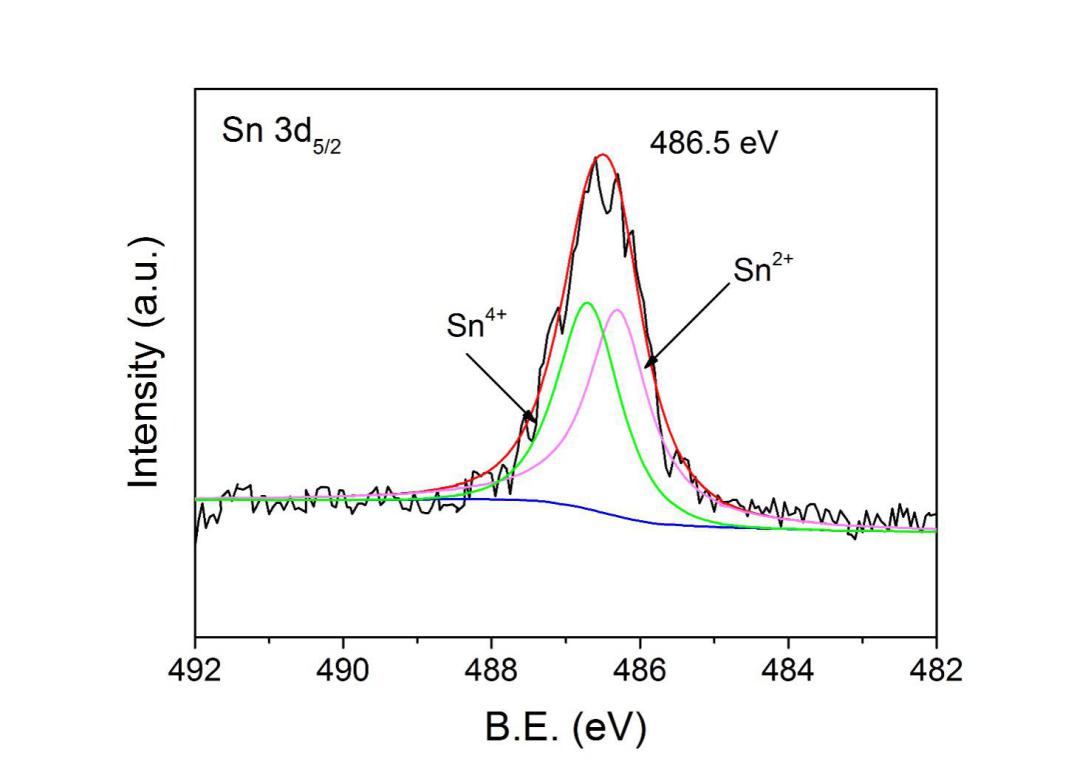


**Figure S14.** XPS spectra of Sn 3d_5/2_ in Sn_1_/CuO.


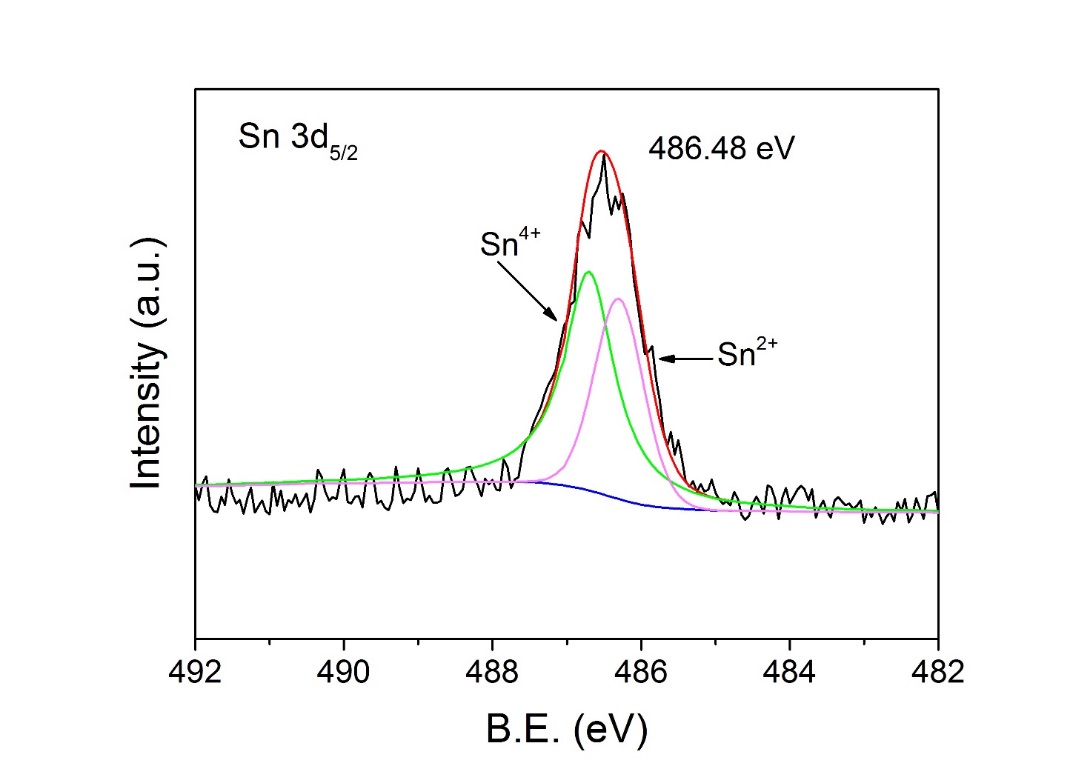


**Figure S15.** XPS spectra of Sn 3d_5/2_ in 0.05Zn_1_-Sn_1_/CuO.


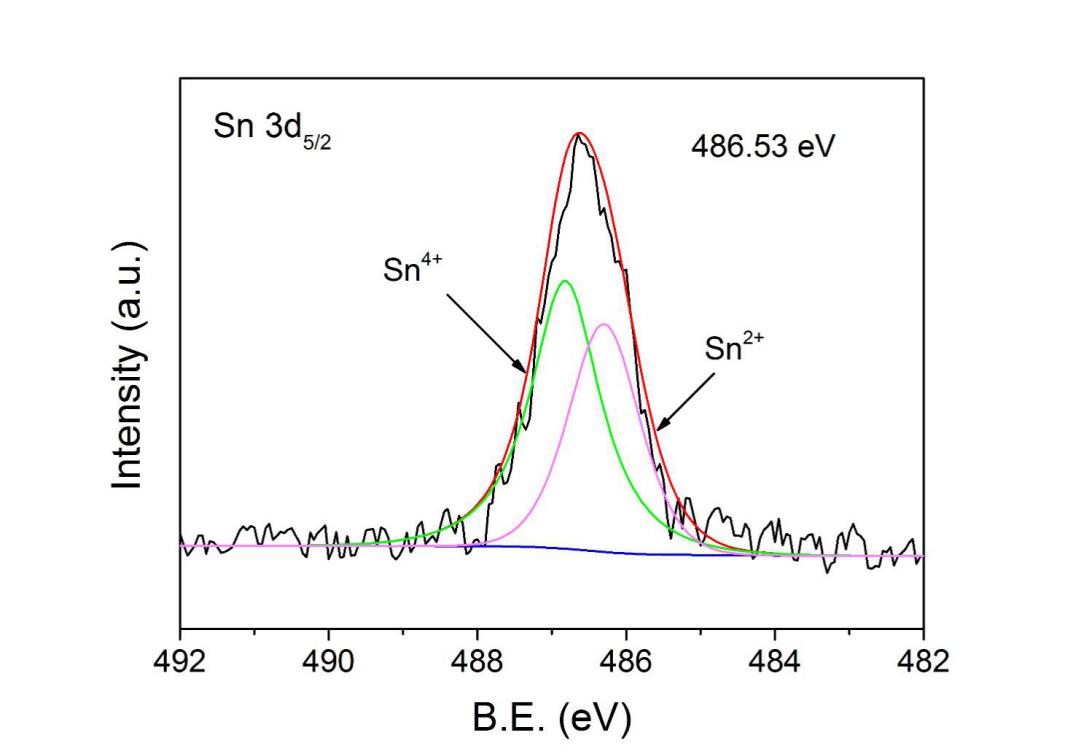


**Figure S16.** XPS spectra of Sn 3d_5/2_ in 0.1Zn_1_-Sn_1_/CuO.


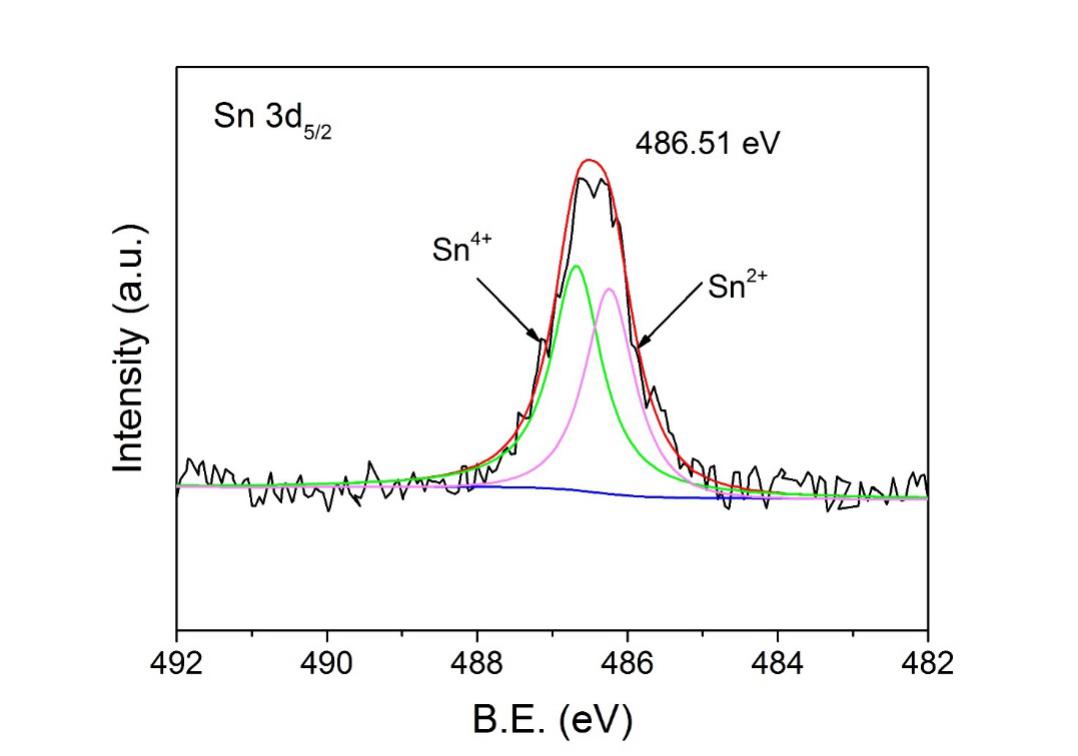


**Figure S17.** XPS spectra of Sn 3d_5/2_ in 0.2Zn_1_-Sn_1_/CuO.


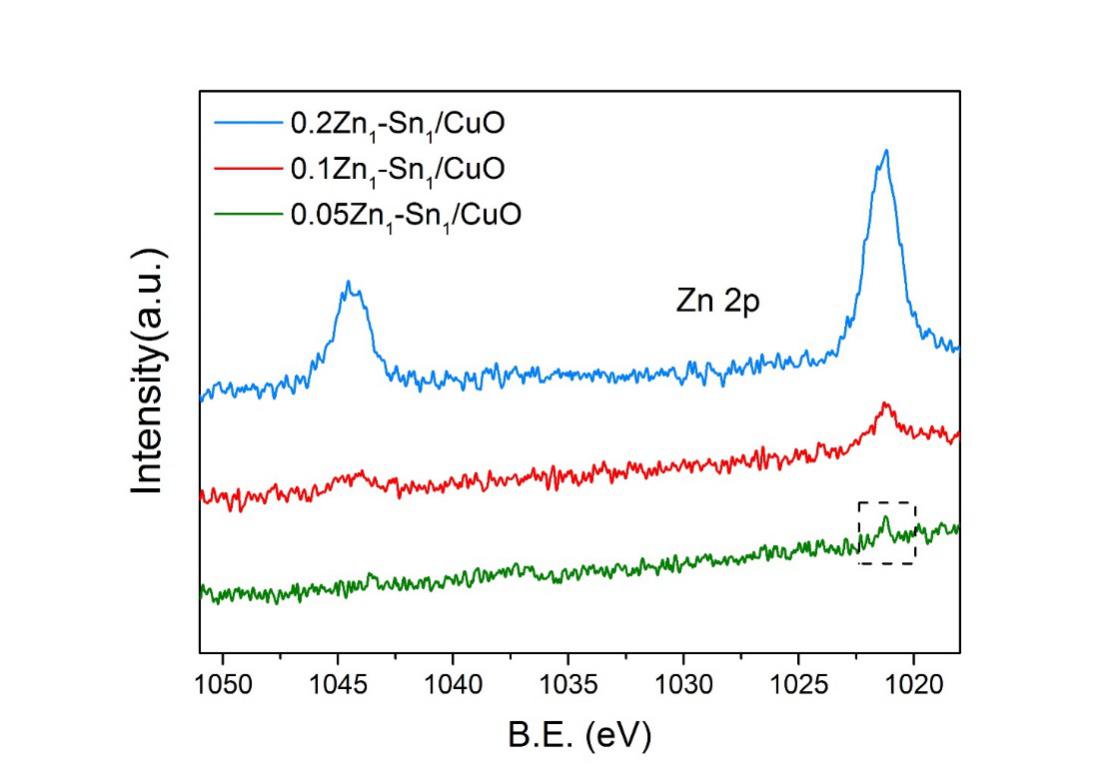


**Figure S18.** XPS spectra of Zn 2p_3/2_ in 0.05Zn_1_-Sn_1_/CuO, 0.1Zn_1_-Sn_1_/CuO, and 0.2Zn_1_-Sn_1_/CuO.

**Scheme S1.** Distribution of the main products in Rochow reaction.

**
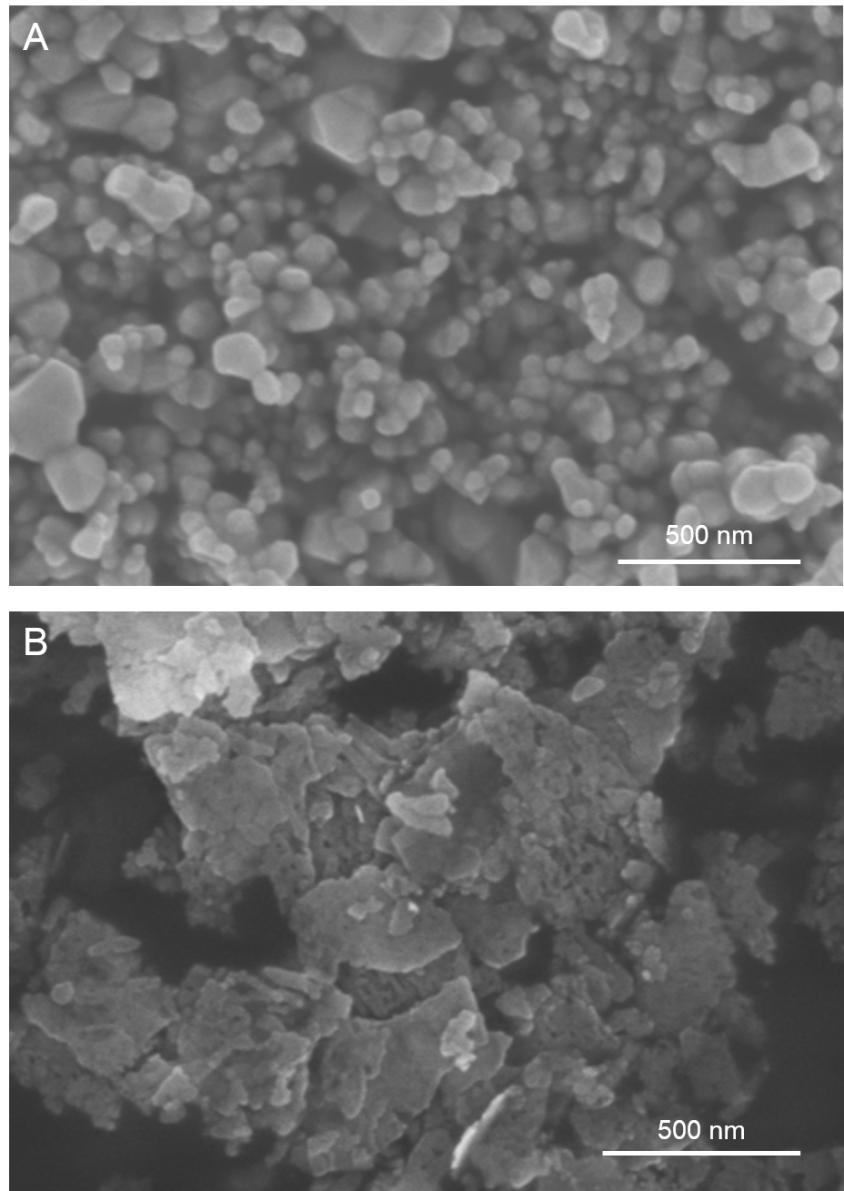
**

**Figure S19.** The SEM images of (A) Sn and (B) Zn nanoparticles.

**
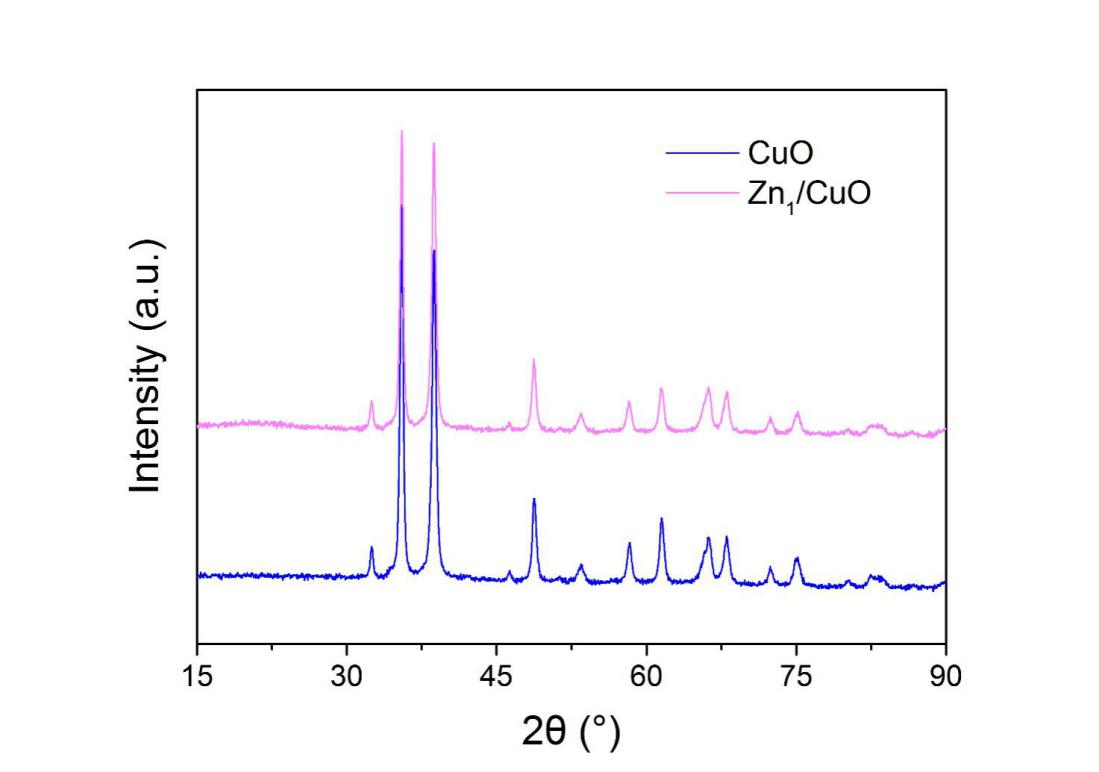
**

**Figure S20.** XRD patterns of CuO and Zn_1_/CuO, in which CuO was used for comparison.


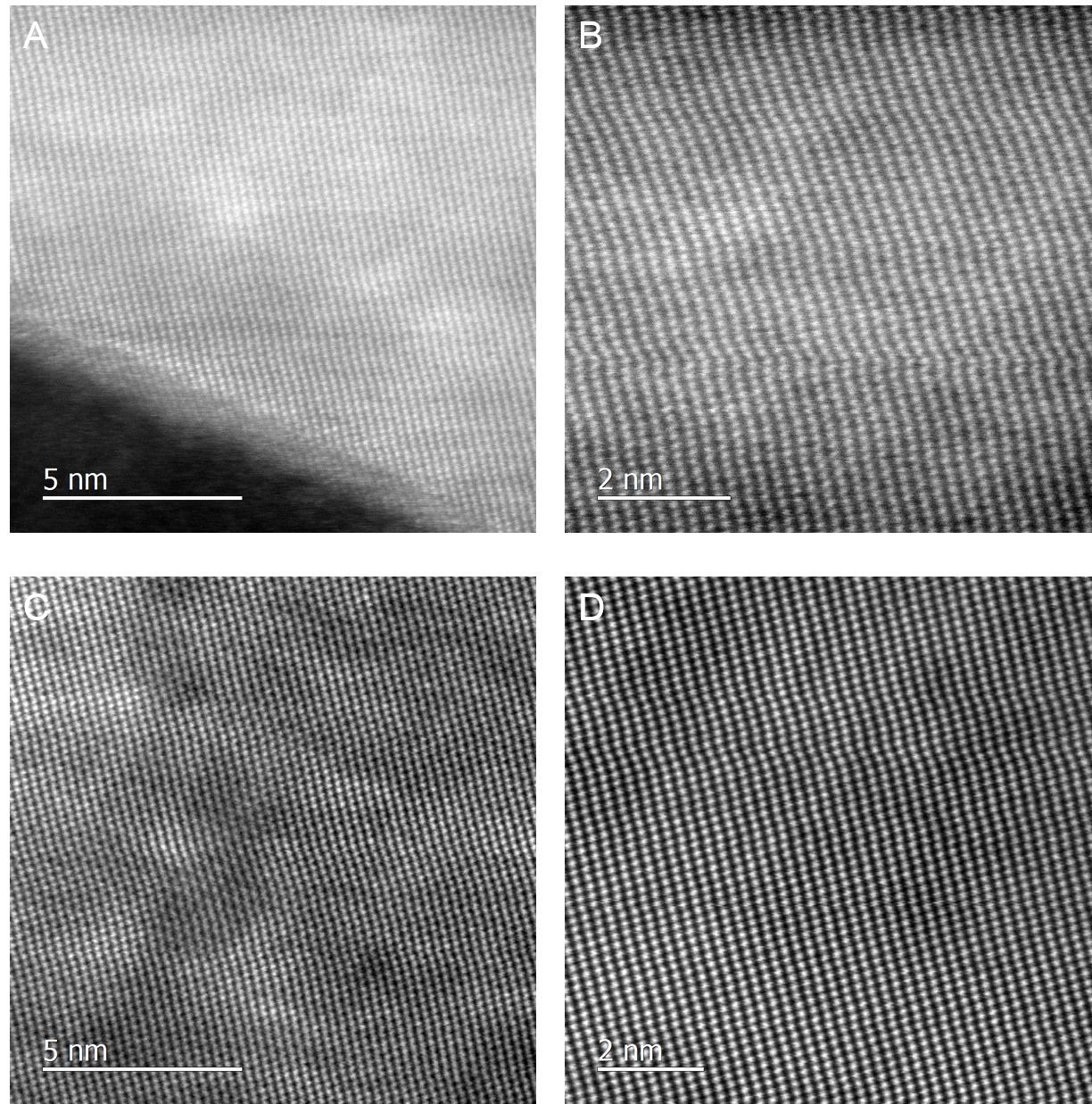


**Figure S21.** AC HAADF-STEM images of (A) and (B) CuO; (C) and (D) Zn_1_/CuO. CuO was used for comparison.


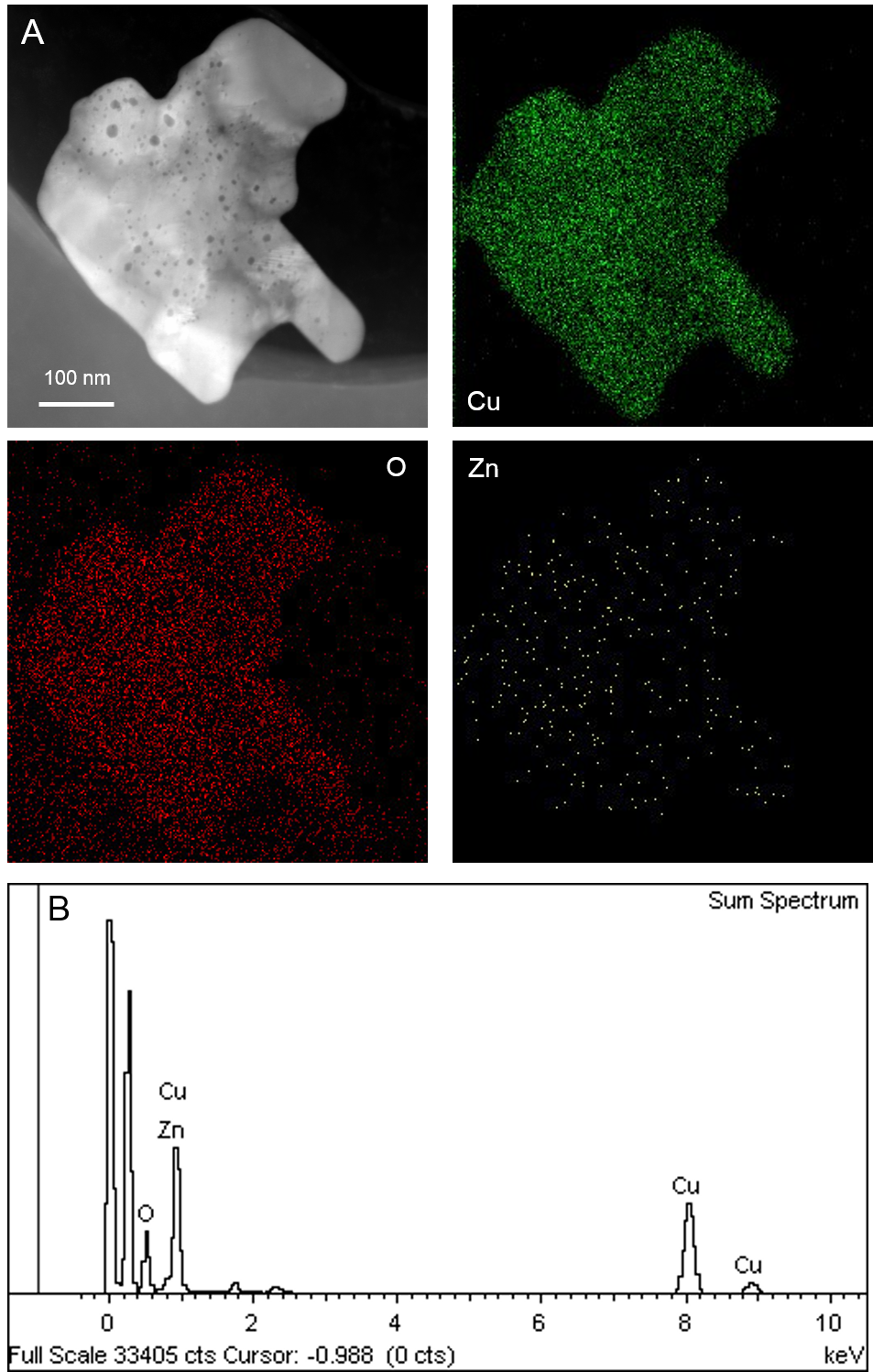


**Figure S22.** (A) HAADF-STEM image and the corresponding EDX mappings as well as (B) the **EDX spectra** of Zn_1_/CuO.

**
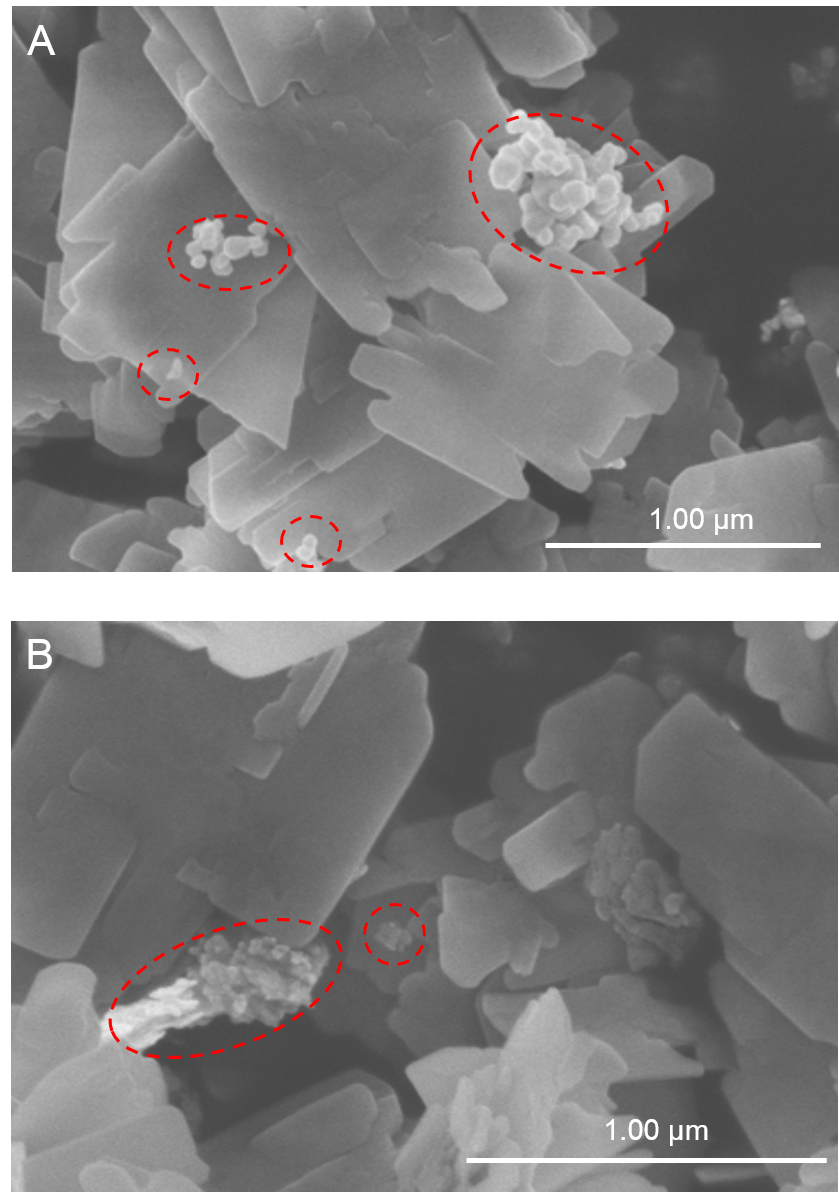
**

**Figure S23.** The SEM images of (A) CuO-5%Sn and (B) CuO-5%Zn. The marked red circles in images A indicate Sn nanoparticles, and the marked red circles in images B indicate Zn nanoparticles.

**Table S3.** Catalytic performance of the samples.

| Entry | Catalyst | Product Selectivity (%) | | | | | | | *C_Si_*  (%) |
| --- | --- | --- | --- | --- | --- | --- | --- | --- | --- |
|  |  | M2 | M1 | M3 | M2H | M1H | LB | HB |  |
| 1 | CuO-5%Sn | 64.1 | 25.8 | 3.7 | 0.8 | 1.2 | 2.8 | 1.6 | 7.2 |
| 2 | CuO-5%Zn | 75.6 | 12.6 | 4.7 | 2.4 | 2.3 | 1.6 | 0.8 | 25.6 |
| 3 | CuO-5%Sn-5%Zn | 77.1 | 11.5 | 4.1 | 2.3 | 1.8 | 2.0 | 1.2 | 27.0 |
| 4 | Sn_1/_CuO-5%Zn | 78.7 | 10.9 | 1.5 | 1.2 | 7.2 | 0 | 0.5 | 28.0 |

Reaction conditions: temperature, 325 °C; time, 24 h; catalyst, 0.5 g; Si 10 g; MeCl gas flow rate, 25 mL min^-1^.

**Table S4.** Catalytic stability data of CuO.

| Time  (h) | Product Selectivity (%) | | | | | | | *C_Si_*  (%) |
| --- | --- | --- | --- | --- | --- | --- | --- | --- |
|  | M2 | M1 | M3 | M2H | MIH | LB | HB |  |
| 6 | 5.3 | 3.3 | 0 | 0 | 0 | 79.4 | 12.0 |  |
| 12 | 3.7 | 2.9 | 0 | 3.4 | 0 | 79.3 | 10.7 |  |
| 18 | 30.7 | 14.4 | 2.3 | 0 | 0 | 48.4 | 4.2 |  |
| 24 | 33.1 | 19.5 | 1.9 | 3.8 | 0.6 | 13.9 | 27.2 | 3 |
| 30 | 0 | 59.9 | 0 | 0 |  | 0 | 40.1 |  |
| 36 | 0 |  |  |  |  |  |  |  |
| 42 | 0 | 1.8 | 0 | 0 | 0 | 94.9 | 3.3 |  |
| 48 | 0 | 28.3 | 0 | 0 | 0 | 33.4 | 38.3 | 5.7 |
| 72 | 0 | 1.5 |  |  |  | 93.7 | 4.8 | 6.9 |

Reaction conditions: temperature, 325 °C; time, 72 h; catalyst, 0.5 g; Si 10 g; MeCl gas flow rate, 25 mL min^-1^.

**Table S5.** Catalytic stability data of Sn_1_/CuO.

| Time  (h) | Product Selectivity (%) | | | | | | | *C_Si_*  (%) |
| --- | --- | --- | --- | --- | --- | --- | --- | --- |
|  | M2 | M1 | M3 | M2H | MIH | LB | HB |  |
| 6 | 6.9 | 0 | 0 | 8.9 | 0 | 76.6 | 7.6 |  |
| 12 | 50.9 | 20.8 | 4.3 | 6.0 | 0 | 15.3 | 2.7 |  |
| 18 | 81.0 | 8.6 | 4.8 | 0.9 | 0 | 4.3 | 0.4 |  |
| 24 | 81.6 | 9.7 | 3.5 | 1.9 | 1.9 | 0.4 | 1.4 | 23.2 |
| 30 | 80.1 | 8.2 | 0 | 0.3 | 0 | 9.3 | 2.1 |  |
| 36 | 74.5 | 8.0 | 0 | 0 | 0 | 17.2 | 0.3 |  |
| 42 | 80.5 | 7.8 | 0 | 0 | 2.1 | 9.6 | 0 |  |
| 48 | 75.2 | 12.9 | 0 | 0 | 3.1 | 7.6 | 1.2 | 38.6 |
| 72 | 70.3 | 18.8 | 0 | 0 | 1.4 | 8.7 | 0.6 | 46.1 |

Reaction conditions: temperature, 325 °C; time, 72 h; catalyst, 0.5 g; Si 10 g; MeCl gas flow rate, 25 mL min^-1^.

**Table S6.** Catalytic stability data of 0.1Zn_1_-Sn_1_/CuO.

| Time  (h) | Product Selectivity (%) | | | | | | | *C_Si_*  (%) |
| --- | --- | --- | --- | --- | --- | --- | --- | --- |
|  | M2 | M1 | M3 | M2H | MIH | LB | HB |  |
| 6 | 7.5 | 7.2 | 0 | 7.1 | 0 | 70.0 | 8.2 |  |
| 12 | 53.3 | 5.0 | 4.4 | 0.7 | 0 | 36.4 | 0.2 |  |
| 18 | 82.6 | 7.3 | 3.7 | 1.3 | 0 | 4.2 | 0.9 |  |
| 24 | 88.7 | 5.2 | 2.8 | 1.5 | 0.1 | 1.2 | 0.5 | 41.6 |
| 30 | 83.8 | 9.8 | 0 | 0 | 0 | 6.2 | 0.2 |  |
| 36 | 81.9 | 9.1 | 0 | 0 | 0 | 8.8 | 0.2 |  |
| 42 | 83.0 | 11.6 | 0 | 0 | 0 | 5.4 | 0 |  |
| 48 | 80.1 | 10.6 | 0 | 0 | 2.4 | 6.4 | 0.5 | 65.5 |
| 72 | 77.6 | 12.8 | 0 | 0 | 1.5 | 7.3 | 0.8 | 71.2 |

Reaction conditions: temperature, 325 °C; time, 72 h; catalyst, 0.5 g; Si 10 g; MeCl gas flow rate, 25 mL min^-1^.


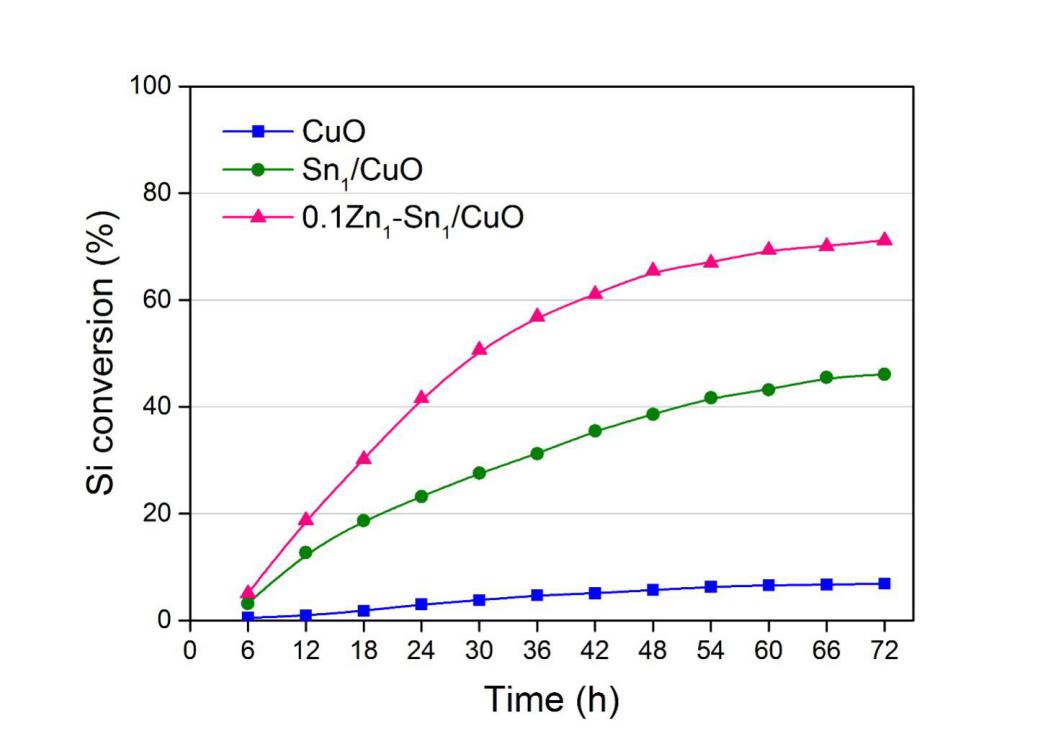


**Figure S24.** The Si conversion as a function of time for CuO, Sn_1_/CuO and 0.1Zn_1_-Sn_1_/CuO in reaction of 72 h.


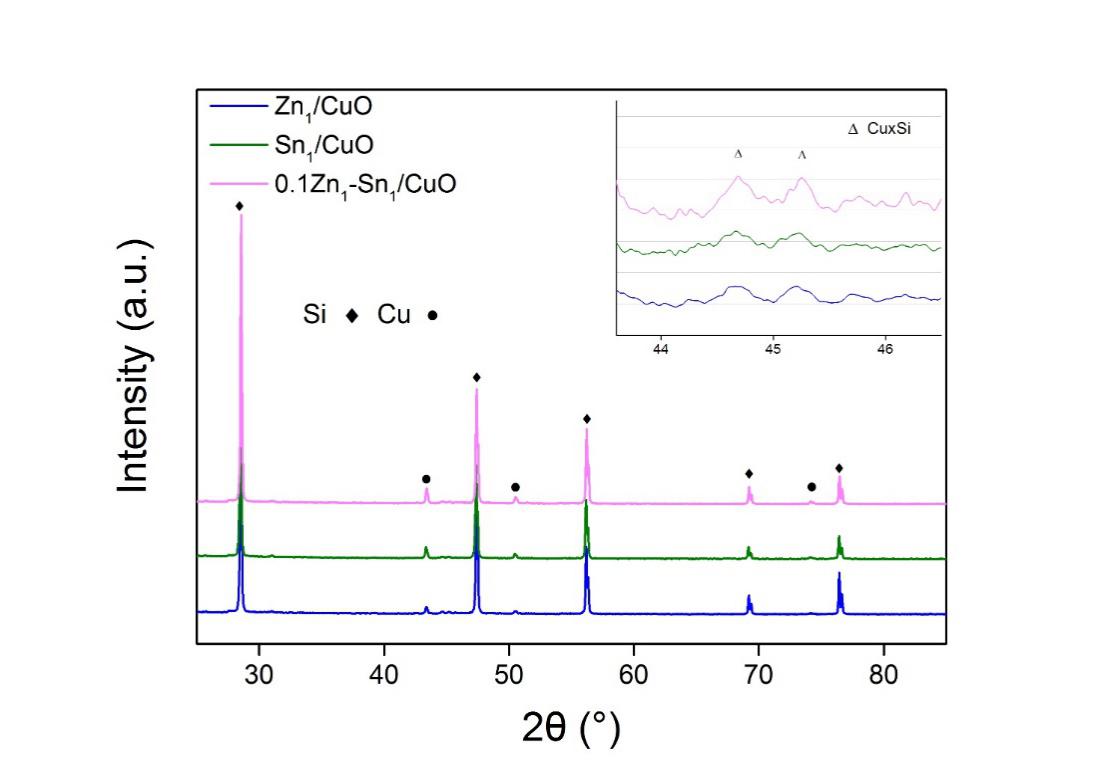


**Figure S25.** XRD patterns of the waste contact masses after a 24 h reaction using catalysts of Zn_1_/CuO, Sn_1_/CuO and 0.1Zn_1_-Sn_1_/CuO. The insert is enlarged view in the 2*θ* angle range of 43.5–46.5°.

**
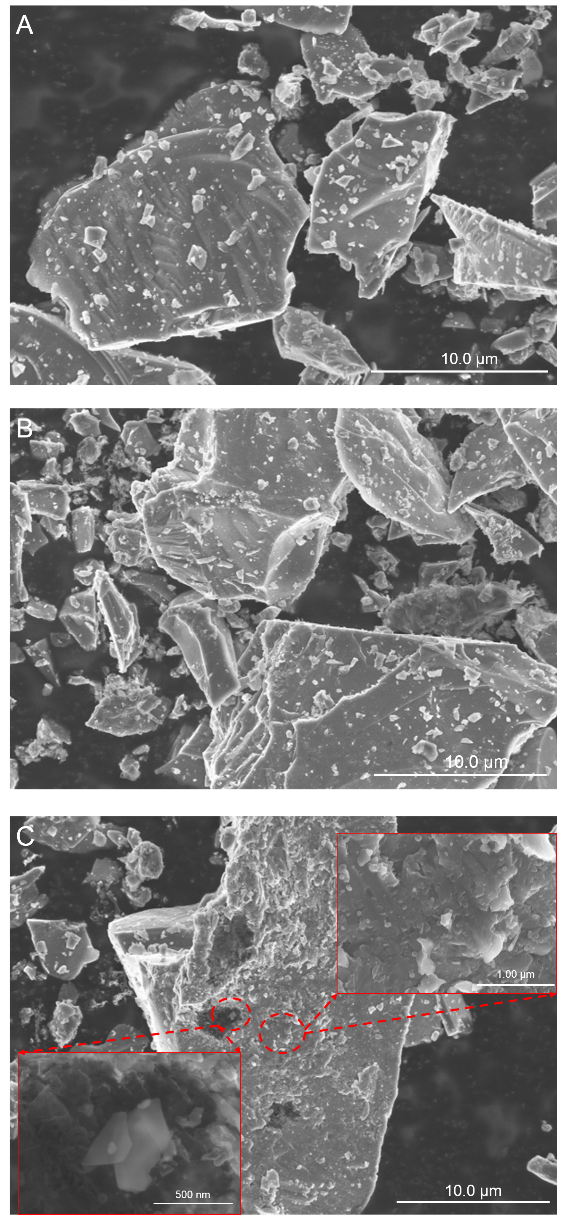
**

**Figure S26.** SEM images of the waste contact masses after a 24 h reaction using catalysts of Zn_1_/CuO, Sn_1_/CuO and 0.1Zn_1_-Sn_1_/CuO. The insert in image C is enlarged SEM image taken from a selected region marked by the red circles. Built-in image in bottom left shows that Zn_1_-Sn_1_/CuO catalyst remain unchanged after reaction, and built-in image in top right shows that smooth Si surface before reaction becomes coarse.

**
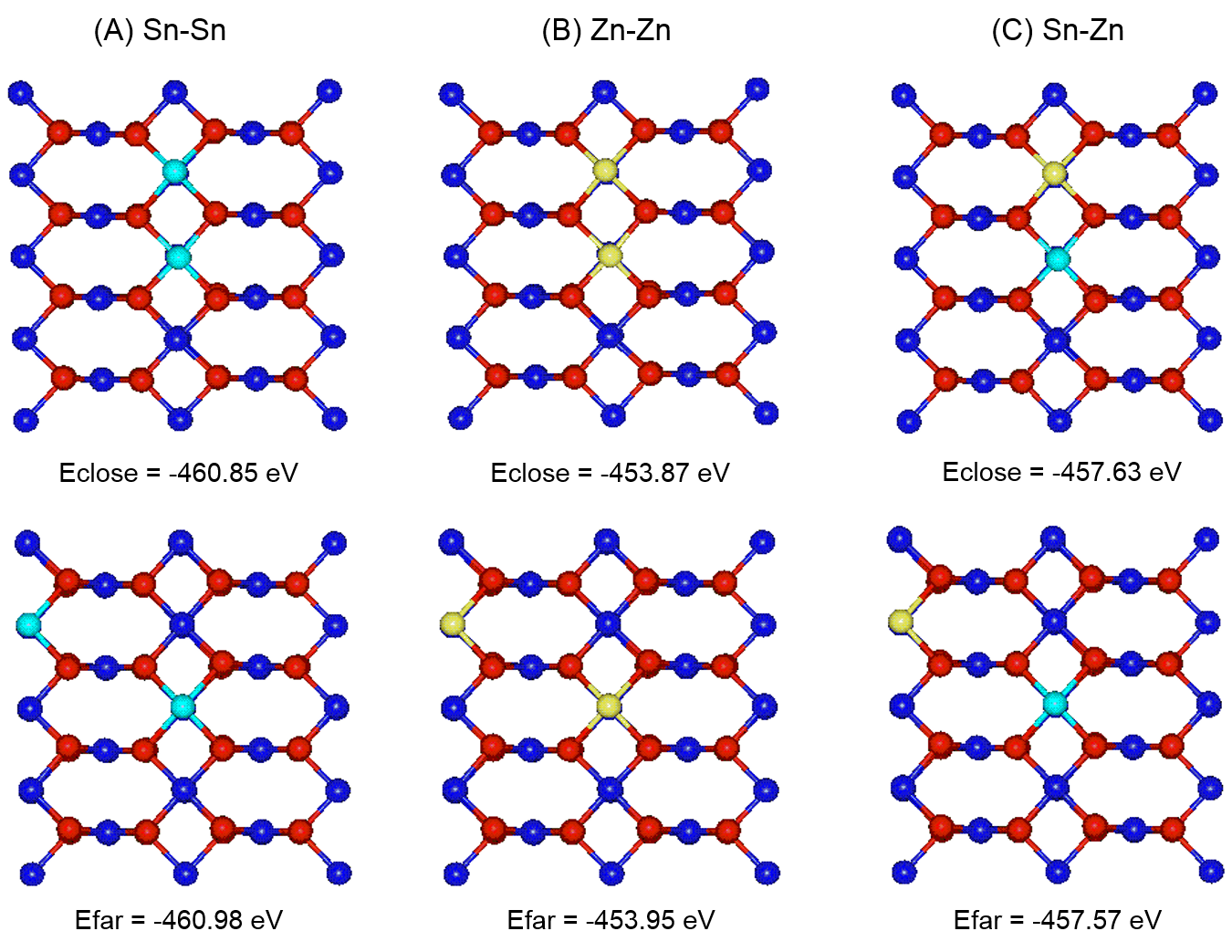
**

**Figure S27.** The energy of (A) Sn-Sn, (B) Zn-Zn, and (C) Sn-Zn interaction on the surface of CuO. Color scheme: O, red; Cu, blue; Sn, bright blue; Zn, yellow. E_close:_ the position is adjacent, E_far_: the position is far away from each other.


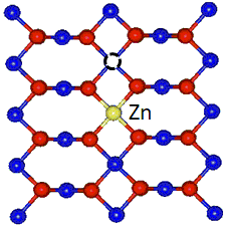


**Figure S28.** The DFT model of Zn-doped CuO(110) surface. Color scheme: O, red; Cu, blue; Zn, yellow.

**Table S7.** The corresponding formation energy of Cu vacancy and adsorption energy of MeCl in Figure 3.

| Sample | E_f_ (V_Cu_)/eV*^a^* | | E_ads_(MeCl)/eV*^b^* |
| --- | --- | --- | --- |
|  | Cu-rich | Cu-poor |  |
| CuO(110)+V_Cu_ | 3.80 | 2.09 |  |
| Sn_1_/CuO(110)+V_Cu_ | 3.02 | 1.31 |  |
| Zn_1_/CuO(110)+V_Cu_ | 3.93 | 2.22 |  |
| CuO(110)+(MeCl)_diss_ |  |  | 0.94 |
| Sn_1_/CuO(110)+(MeCl)_diss_ |  |  | -1.32 |
| Zn_1_/CuO(110)+(MeCl)_diss_ |  |  | 0.08 |
| Zn_1_-Sn_1_/CuO(110)+(MeCl)_diss_ |  |  | -1.07 |

*^a^* E_f_ (V_Cu_): the formation energy of Cu vacancy; *^b^* E_ads_(MeCl): the adsorption energy of MeCl, and positive value indicates unstable adsorption while negative value indicates stable adsorption.


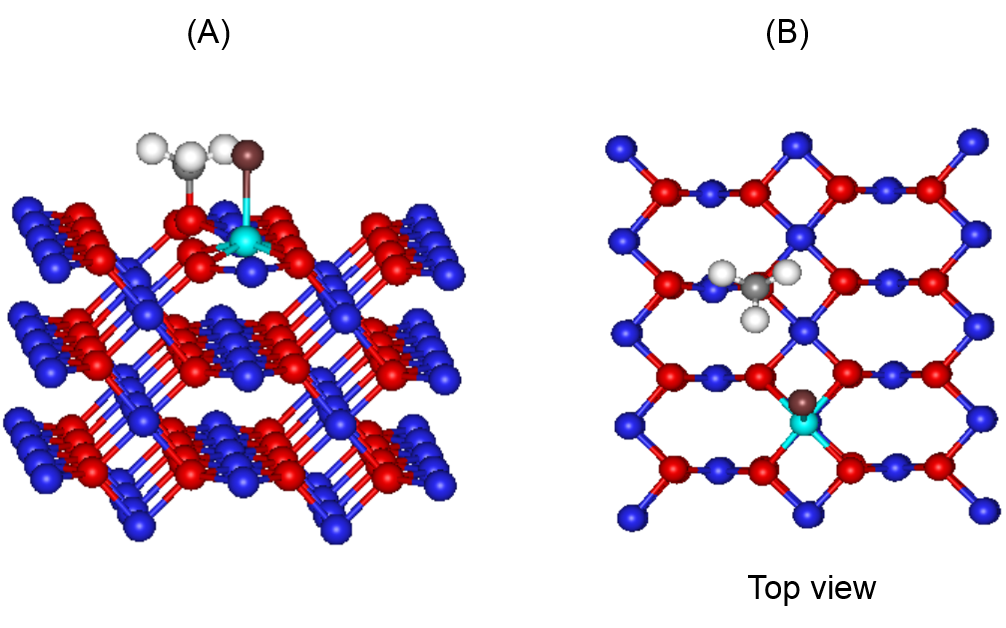


**Figure S29.** (A) Optimized surface atomic structures and (B) the top view with MeCl dissociative adsorption on Sn doped CuO(110). Color scheme: O, red; Cu, blue; C, gray; H, white; Cl, brown; Sn, bright blue.


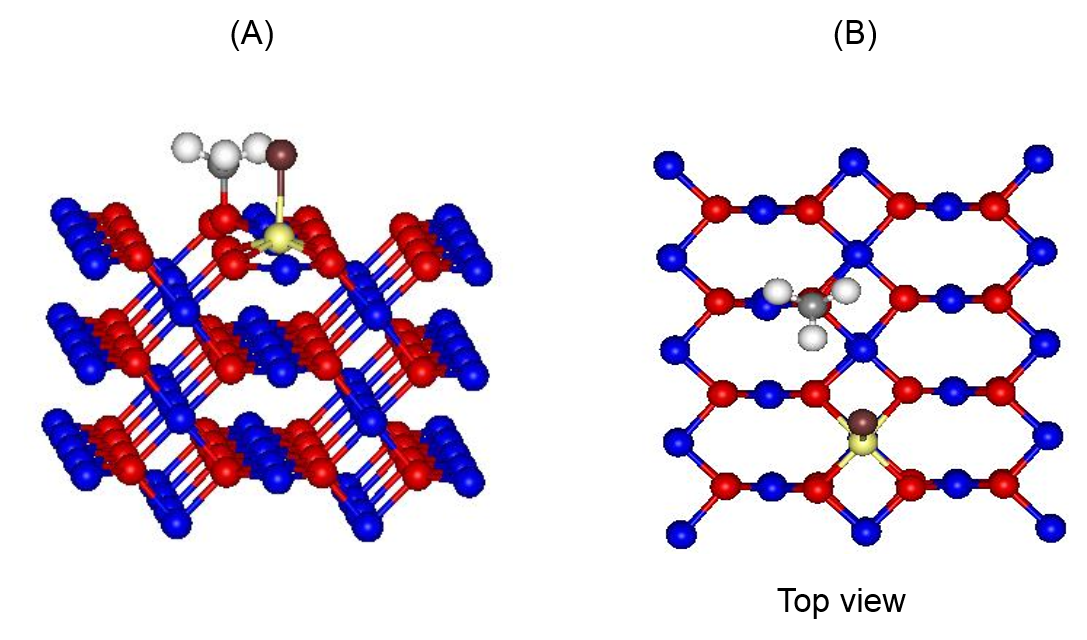


**Figure S30.** (A) Optimized surface atomic structures and (B) the top view with MeCl dissociative adsorption on Zn doped CuO(110). Color scheme: O, red; Cu, blue; C, gray; H, white; Cl, brown; Zn, yellow.

**Table S8.** The number of electrons on 48 Cu atoms.

|  | CuO | Sn_1_/CuO | Zn_1_/CuO | Sn_1_/CuO+V_Cu_ | Zn_1_-Sn_1_/CuO |
| --- | --- | --- | --- | --- | --- |
|  | 10.173 | 10.174 | 10.173 | 10.171 | 10.174 |
|  | 10.159 | 10.16 | 10.159 | 10.176 | 10.158 |
|  | 10.173 | 10.174 | 10.173 | 10.175 | 10.176 |
|  | 10.159 | 10.16 | 10.173 | 10.163 | 10.187 |
|  | 10.173 | 10.175 | 10.173 | 10.18 | 10.202 |
|  | 10.159 | 10.159 | 10.159 | 10.179 | 10.149 |
|  | 10.173 | 10.18 | 10.177 | 10.17 | 10.16 |
|  | 10.159 | 10.175 | 10.158 | 10.174 | 10.177 |
|  | 10.173 | 10.174 | 10.174 | 10.203 | 10.194 |
|  | 10.159 | 10.171 | 10.167 | 10.173 | 10.176 |
|  | 10.173 | 10.174 | 10.173 | 10.157 | 10.163 |
|  | 10.159 | 10.159 | 10.158 | 10.157 | 10.173 |
|  | 10.173 | 10.158 | 10.155 | 10.275 | 10.24 |
|  | 10.159 | 10.199 | 10.213 | 10.158 | 10.159 |
|  | 10.173 | 10.159 | 10.159 | 10.201 | 10.201 |
|  | 10.159 | 10.201 | 10.201 | 10.03 | 10.031 |
|  | 10.201 | 10.031 | 10.03 | 10.196 | 10.196 |
|  | 10.03 | 10.196 | 10.196 | 10.221 | 10.221 |
|  | 10.195 | 10.221 | 10.221 | 10.201 | 10.201 |
|  | 10.221 | 10.201 | 10.201 | 10.03 | 10.03 |
|  | 10.201 | 10.031 | 10.03 | 10.195 | 10.195 |
|  | 10.03 | 10.194 | 10.195 | 10.22 | 10.218 |
|  | 10.195 | 10.221 | 10.221 | 10.201 | 10.201 |
|  | 10.221 | 10.201 | 10.201 | 10.03 | 10.031 |
|  | 10.201 | 10.031 | 10.03 | 10.194 | 10.195 |
|  | 10.03 | 10.195 | 10.196 | 10.221 | 10.221 |
|  | 10.195 | 10.222 | 10.221 | 10.199 | 10.2 |
|  | 10.221 | 10.201 | 10.201 | 10.03 | 10.031 |
|  | 10.201 | 10.031 | 10.03 | 10.161 | 10.189 |
|  | 10.03 | 10.195 | 10.195 | 10.22 | 10.221 |
|  | 10.195 | 10.22 | 10.221 | 10.201 | 10.201 |
|  | 10.221 | 10.201 | 10.201 | 10.031 | 10.03 |
|  | 10.201 | 10.032 | 10.03 | 10.195 | 10.197 |
|  | 10.03 | 10.195 | 10.195 | 10.222 | 10.219 |
|  | 10.195 | 10.221 | 10.221 | 10.201 | 10.201 |
|  | 10.221 | 10.201 | 10.201 | 10.03 | 10.031 |
|  | 10.201 | 10.031 | 10.03 | 10.197 | 10.198 |
|  | 10.03 | 10.194 | 10.195 | 10.221 | 10.221 |
|  | 10.195 | 10.222 | 10.221 | 10.201 | 10.201 |
|  | 10.221 | 10.2 | 10.2 | 10.031 | 10.031 |
|  | 10.201 | 10.031 | 10.03 | 10.215 | 10.218 |
|  | 10.03 | 10.191 | 10.195 | 10.222 | 10.222 |
|  | 10.195 | 10.221 | 10.221 | 10.201 | 10.201 |
|  | 10.221 | 10.201 | 10.201 | 10.03 | 10.031 |
|  | 10.201 | 10.031 | 10.03 | 10.199 | 10.197 |
|  | 10.03 | 10.195 | 10.195 | 10.221 | 10.221 |
|  | 10.195 | 10.221 | 10.221 | 2.259 | 2.275 |
|  | 10.221 | 2.178 | 10.966 |  | 10.989 |
| Total*^a^* | 467.488 | 467.551 | 467.544 | 467.679 | 467.69 |

*^a^* the total number of electrons on 46 Cu atoms except for the positions where Sn and Zn are replaced. It shows that in the Sn-Zn pair doped case Cu atoms have the most valence electrons.
